# Tissue Engineered 3D Constructs for Volumetric Muscle Loss

**DOI:** 10.1007/s10439-024-03541-w

**Published:** 2024-07-31

**Authors:** Sonal Gahlawat, Doga Oruc, Nikhil Paul, Mark Ragheb, Swati Patel, Oyinkansola Fasasi, Peeyush Sharma, David I. Shreiber, Joseph W. Freeman

**Affiliations:** Department of Biomedical Engineering, Rutgers University–New Brunswick, Piscataway, NJ USA

**Keywords:** Skeletal muscle regeneration, Volumetric muscle loss, Biomaterials, Tissue engineering, Scaffolds, Decellularization, Electrospinning, Hydrogels

## Abstract

Severe injuries to skeletal muscles, including cases of volumetric muscle loss (VML), are linked to substantial tissue damage, resulting in functional impairment and lasting disability. While skeletal muscle can regenerate following minor damage, extensive tissue loss in VML disrupts the natural regenerative capacity of the affected muscle tissue. Existing clinical approaches for VML, such as soft-tissue reconstruction and advanced bracing methods, need to be revised to restore tissue function and are associated with limitations in tissue availability and donor-site complications. Advancements in tissue engineering (TE), particularly in scaffold design and the delivery of cells and growth factors, show promising potential for regenerating damaged skeletal muscle tissue and restoring function. This article provides a brief overview of the pathophysiology of VML and critiques the shortcomings of current treatments. The subsequent section focuses on the criteria for designing TE scaffolds, offering insights into various natural and synthetic biomaterials and cell types for effectively regenerating skeletal muscle. We also review multiple TE strategies involving both acellular and cellular scaffolds to encourage the development and maturation of muscle tissue and facilitate integration, vascularization, and innervation. Finally, the article explores technical challenges hindering successful translation into clinical applications.

## Introduction

Volumetric muscle loss (VML) is a debilitating condition characterized by the substantial loss of skeletal muscle volume resulting from severe trauma, crush injuries, and associated surgical procedures, ultimately leading to structural and functional impairment [1, 2]. When the loss of skeletal muscle exceeds 20%, the innate capacity for repair and regeneration is permanently compromised [3]. While VML injuries are prevalent in both civil and military populations, the latter experience a disproportionately higher frequency, constituting approximately 50% of combat-related injuries [4]. While VML is challenging to track in the civilian population due to the lack of billable therapy, military personnel, particularly those with type-III open tibia fractures involving severe bone and soft-tissue injuries, report a 65% incidence of permanent disability leading to medical retirement [5, 6].

The physiological repair framework for skeletal muscle involves basal lamina and satellite cells, which are capable of proliferation and differentiation to generate or repair myofibers. In VML, extensive tissue and vascularity loss impede the regenerative capacity of skeletal muscles, resulting in chronic tissue loss, strength deficits, and disability [7, 8]. While VML severity is often reported in terms of percent mass loss, the functional deficits observed in clinics, such as loss of muscle strength and restricted range of motion, consistently surpass the predicted magnitude [6].

**Fig. 1:**
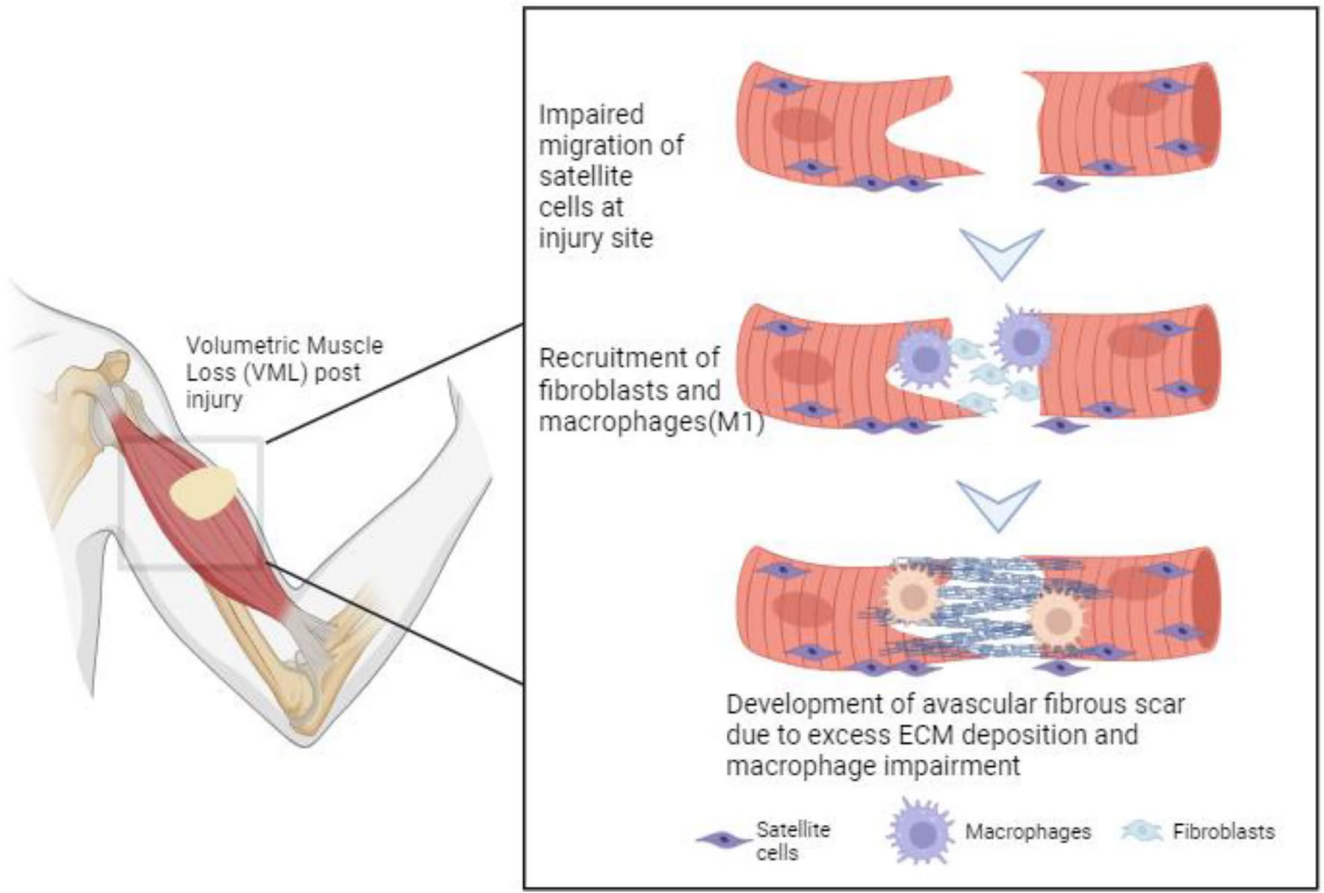
Schematic representation of the challenges associated with VML. Significant mass loss due to injury impairs the migration of satellite cells at the injury site, leading to lack of regeneration cues and metabolic activity. VML also results in the recruitment of fibroblasts at the injury site, causing deposition of unorganized ECM that lacks orientation and vasculature. The lack of phenotypic switching of macrophages into M2 prolongs the inflammatory environment at the injury site. Created with Biorender.com

A VML event triggers a cascade of intertwined pathophysiological responses across injured and uninjured cells, affecting various tissue types, including muscle, bone, tendons, nerves, and blood vessels (Fig. 1). Following primary injury, cellular damage and alterations in the tissue structure initiate an inflammatory response, where macrophages play a crucial role in muscle repair [9]. Normal regeneration of skeletal muscles involves a slow, energy-intensive process, including robust inflammatory cell deployment followed by rapid migration and proliferation of muscle progenitor cells, i.e., satellite cells, and generation of vasculature and extracellular matrix (ECM). Specifically, the dormant tissue-resident macrophages undergo a phenotypic switch from M1 “inflammatory” to M2 “anti-inflammatory,” supporting myogenic progenitor differentiation and myofiber growth [10–12]. However, in VML, prolonged macrophage presence in an intermediate phenotype leads to excessive ECM accumulation without generating the required muscle fibers and subsequently to non-functional scar formation, also known as pathologic fibrosis [13, 14]. Loss of native muscle scaffolding further restricts satellite cell migration, hindering vascular network formation and impairing motor neuron terminal innervation typical of neuro-muscular junctions (NMJs) [15, 16]. The resulting deficit in tissue volume leads to decreased oxidative metabolic activity and a subsequent metabolic imbalance due to the loss of mitochondria-rich skeletal muscle cells. Mitochondria function as calcium sinks, and an elevation in cytosolic calcium levels overwhelms mitochondrial calcium, posing a threat to mitochondrial function and cell viability and further contributing to skeletal muscle loss affected by VML [17]. The pathophysiological events post-VML make natural healing highly improbable, potentially leading to comorbidities and eventual amputation. VML induces a range of functional deficits impacting an individual’s well-being, including a substantial reduction in strength, compromised muscle function, and restricted range of motion. These impairments severely affect daily activities, hinder mobility, and curtail engagement in physically demanding tasks [2].

The treatment and recovery of VML pose formidable challenges due to the intricate mechanisms of muscle regeneration. Currently, no standard of care for VML injuries can fully restore function to injured skeletal muscles. The gold standard involves soft tissue reconstruction using functional free muscle transfer and/or fasciocutaneous flaps. While promising, this approach requires a highly skilled surgical team and proper patient selection, with factors like blood supply, muscle architecture, and donor site morbidity dictating success [18–21]. Even in successful functional muscle transfer cases, there is a potential for partial graft integration with the host vascular and neural networks, resulting in persistent functional deficits [7]. Physical therapy is consistently recommended to facilitate healing and fortify the remaining muscle. Advanced bracing strategies, such as carbon fiber-based braces, can be custom-designed to physically support the patient in restoring partial or total functionality [22]. While the braces have the advantage of being a non-surgical treatment option, these are relatively expensive solutions and are only available for partial and total muscle loss below the knees, with no alternative available for muscle loss above the knee [1]. Rehabilitation and physical exercises have shown encouraging results in functional recovery after VML injury [23]. However, improved functional outcomes were due to increased force transmission induced by physical rehabilitation and from the morphological adaptations or de novo tissue formation [24]. Further, these results did not translate into long-term improvement when investigated as a standalone treatment [24]. While both bracing strategies and physical rehabilitation show some promise, they often result in prolonged functional deficits and compromised quality of life in VML patients.

With its remarkable regenerative capacity, skeletal muscle relies on satellite cells for repair and regeneration after injury. However, in VML, the absence of regenerative cues from satellite cells leads to fibrosis, where muscle fibers are replaced by fibrous scar tissue [25]. Given the complex hierarchical structure of skeletal muscles involving connective, nerve, and vascular tissue, a combination treatment approach is essential for enhancing muscle regeneration, reducing fibrosis, and promoting functional recovery [26]. Tissue engineering (TE) strategies emerge as a promising avenue to stabilize VML defects through revascularization and neuromuscular tissue regeneration. TE scaffolds, from various natural and synthetic biomaterials, can offer structural support and stimulate cellular activity for functional tissue regeneration.

## Design Criteria

**Fig. 2:**
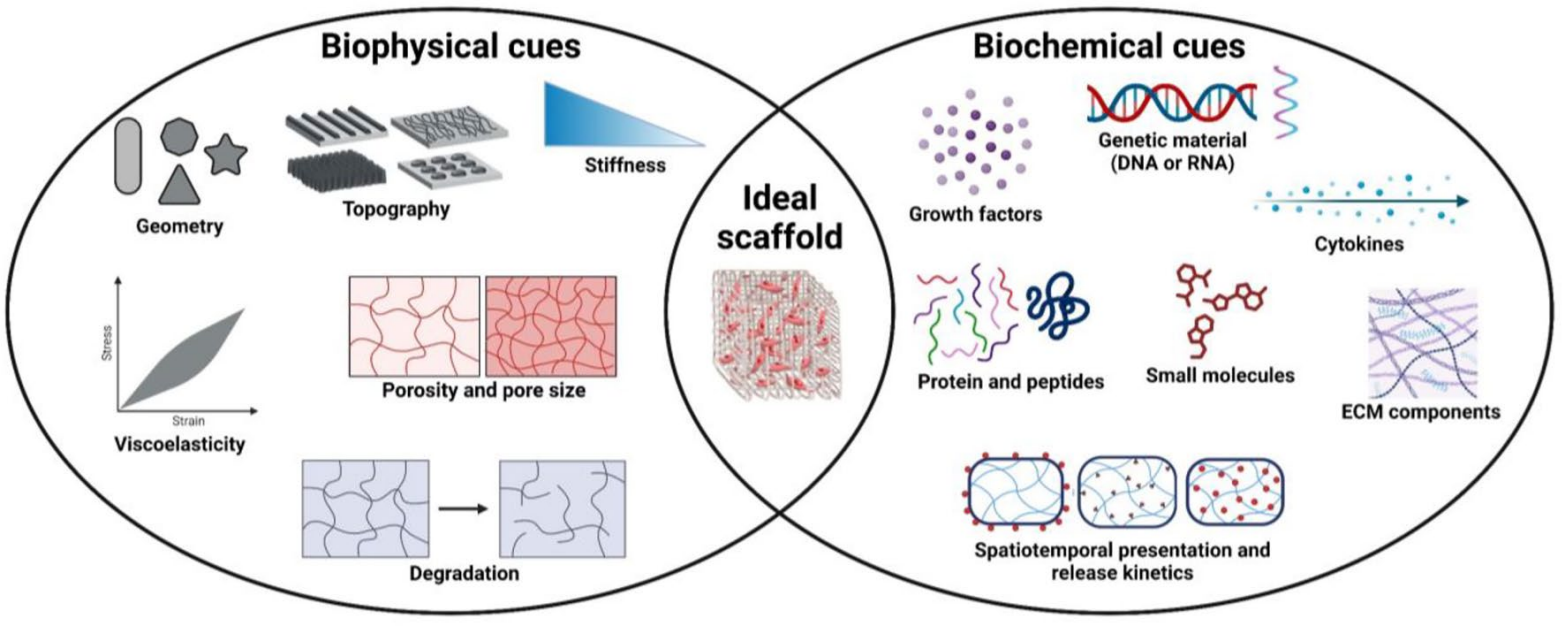
Biophysical and biochemical cues used for designing biomaterials in skeletal muscle TE. Biophysical and biochemical cues can be used synergistically for designing biomaterials for skeletal muscle TE. Biophysical cues include geometry, topography, stiffness and viscoelasticity, porosity and pore size, and degradation. Biochemical cues include spatiotemporal delivery of genetic material, growth factors, small molecules, cytokines, proteins and peptides, and ECM components. In combination, biophysical and biochemical cues provide a biomimetic architecture to guide cellular functions and tissue regeneration. Created with Biorender.com

An ideal TE scaffold for skeletal muscle regeneration has four primary objectives: (1) facilitate cell alignment; (2) promote functional muscle regeneration; (3) stimulate vascularization and innervation; and (4) prevent scar tissue formation [27]. The three-dimensional (3D) scaffold must emulate the in vivo microenvironment of skeletal muscle, providing a biomimetic architecture to guide cellular functions and tissue regeneration. A scaffold should offer biophysical and biochemical cues that mimic native tissue composition, architecture, mechanics, and bioactive signaling, which are essential for satellite cells to differentiate and organize into functional muscle tissue [27, 28]. Key biophysical cues include topography, porosity, and mechanics, while biochemical cues involve spatial and temporal control over the presentation of bioactive molecules, such as growth factors, cell adhesion molecules, cytokines (Fig. 2) [27]. Biocompatibility is critical to avoid toxicity and foreign body responses, which could lead to tissue fibrosis, encapsulation, and graft failure.

Given that a TE scaffold serves as a template for tissue regeneration and guides the formation of new tissue locally, it is crucial to tailor its properties, such as stiffness, degradation, and porosity, for proper integration with the host tissue. An ideal scaffold for treating VML injuries should exhibit a stiffness similar to that of skeletal muscles, with Young’s modulus of approximately 8–17 kPa [29, 30]. Myoblasts cultured on substrates with Young’s modulus of about 12 kPa demonstrated significantly higher actin/myosin striations compared to substrates with lower or higher stiffness that showed no striations, highlighting the role between scaffold’s stiffness and a more functional and mature cellular phenotype [29]. The mechanical stiffness of the scaffold regulates gene expression of anchorage-dependent cells, such as myoblasts, to influence cell adhesion, proliferation, and differentiation [29, 31]. Scaffold biodegradation is another critical factor determining the success of skeletal muscle regeneration. The rate of scaffold biodegradation should match the rate of skeletal muscle regeneration and new tissue formation [30]. While rapid degradation may result in open spaces filled by scar tissue, compromising the tissue regeneration process and mechanical properties of the scaffold, a slow degradation rate can trigger an immune response, fibrosis, and encapsulation of the scaffold, necessitating surgical intervention [32]. Porosity and pore size are additional biophysical cues for TE scaffolds that impact cellular infiltration, vascularization, mechanical properties, and scaffold degradation. Balancing porosity, pore size, and mechanical properties is crucial for designing an optimal scaffold for skeletal muscle regeneration because of their direct implications on cellular infiltration, vascularization, mechanical properties, and scaffold degradation. Open porous and interconnected networks allow scaffold infiltration by cells, followed by their proliferation and migration for vascularization. This guides the formation of new tissues, which is crucial for successful integration between the scaffold and surrounding tissue [33]. In addition, the porous network enables the diffusion of oxygen and nutrients and the removal of waste necessary for cell survival. However, high porosity can compromise the scaffold’s mechanical properties and structural stability. Therefore, a balance between porosity, pore size, and mechanical property should exist for designing an optimal scaffold for regenerating skeletal muscles.

Incorporating biochemical cues in skeletal muscle TE involves integrating bioactive molecules into the scaffold to control and regulate cellular functions, including adhesion, proliferation, migration, and differentiation. Bioactive molecules include proteins, peptides, growth factors, cytokines, small molecules, and genetic materials (such as cDNA and mRNA). The scaffold design should ensure the spatiotemporal presentation of these bioactive molecules, along with controlled release kinetics and local concentrations [27]. Maintaining the native conformation of bioactive molecules within the scaffold is crucial for regulating their bioactivity and preventing degradation during release [30]. Bioactive molecules can be incorporated into the scaffold through covalent coupling, ionic interactions, or physical entrapment, allowing localized and sustained release at the injury site [34].

## Scaffold Composition

### Natural vs. Synthetic Biomaterial

Scaffolds designed for skeletal muscle regeneration can be crafted from various polymers, including synthetic, natural, and combinations of the two. Each biomaterial category has its own advantages and drawbacks (Table 1). Natural biomaterials, sourced from proteins, DNA, polysaccharides, etc., offer the benefits of enhanced biocompatibility and biodegradability and low cytotoxicity when generally compared to synthetic materials. Moreover, these biomaterials can include native signaling molecules that facilitate cell adhesion, proliferation, migration, and differentiation, along with native functional groups that enable the easy conjugation of bioactive molecules. Natural biomaterials include collagen, gelatin, fibrin, alginate, silk fibroin, hyaluronic acid (HA), decellularized extracellular matrix (dECM), and chitosan. Natural biomaterials may exhibit mechanical weakness, lack precision and tunability in scaffold creation, and are subject to batch-to-batch variability despite their biocompatibility.

**Table 1:** Advantages and disadvantages of natural and synthetic biomaterials

Type of biomaterial	Examples	Advantages	Disadvantages
Natural	Collagen, gelatin, fibrin, alginate, silk fibroin, hyaluronic acid (HA), decellularized ECM (dECM), chitosan	– Biocompatible – Biodegradable – Low cytotoxicity – Contain native signaling molecules that promote cell adhesion, proliferation, migration, and differentiation	– Mechanically weak – Offer less precision and tunability for creating scaffolds – Subject to batch-to-batch variability
Synthetic	Polyethylene glycol (PEG), polypropylene (PP), polylactic acid (PLA or PLLA), polycaprolactone (PCL), polyglycolic acid (PGA), and poly-lactic-co-glycolic acid (PLG or PLGA)	– Inexpensive – Consistent – Offer high precision for creating scaffolds – Offer tunable degradation rates, porosity, and mechanical properties	– Limited compatibility, resulting in an inflammatory response following implantation – Require the conjugation of bioactive molecules and their release for regulating cellular function

In contrast, relative to natural polymers, synthetic biomaterials are cost-effective, consistent, and easily fashioned into precise 3D structures. These biomaterials are amenable to engineering tunable degradation rates, porosity, and mechanical properties. Some synthetic biomaterials, such as polypyrrole, polyaniline, and carbon nanotubes, also possess electrical conductivity, enhancing their potential for engineering skeletal muscles, cardiac muscles, and smooth muscle cells [35]. Common examples of synthetic biomaterials in TE include polyethylene glycol (PEG), polypropylene (PP), polylactic acid (PLA), polycaprolactone (PCL), polyglycolic acid (PGA), and poly-lactic-co-glycolic acid (PLG or PLGA). While synthetic biomaterials offer numerous advantages, they lack bioactive signaling molecules and necessitate the conjugation and release of bioactive molecules to regulate cellular function. Natural and synthetic biomaterials can be employed independently or in combination to formulate an ideal scaffold containing biophysical and biochemical cues for skeletal muscle tissue regeneration.

### Cells

Scaffolds designed for skeletal muscle TE can be utilized independently or in conjunction with cells. Among the commonly employed cell types are satellite cells, myoblasts, muscle-derived precursor cells (MDPCs), perivascular stem cells, adipose-derived stem cells, induced pluripotent stem cells (iPSCs), and mesenchymal stem cells (MSCs) [36, 37]. The ideal cell population for TE scaffolds should be readily available, possess a high proliferative capacity in vitro for scaling up, and demonstrate the ability to differentiate into mature myofibers [28]. The cell source is another significant factor; autologous cells are non-immunogenic and highly suitable for clinical applications, whereas allogeneic and xenogeneic cell sources necessitate immunosuppressive drugs to prevent rejection.

Satellite cells, primarily responsible for skeletal muscle repair and regeneration, are widely used in skeletal muscle TE applications [38]. However, the heterogeneity within the satellite cell population, even from the same source, necessitates advanced purification methods and limits their practicality [32]. This heterogeneity affects their proliferation kinetics and differentiation abilities in vitro and in vivo [39]. Moreover, the continued expansion of satellite cells in vitro leads to senescence and reduced regenerative capacity, further limiting their utility in clinical applications [40]. Myoblasts and MDPCs also exhibit myogenic potential but face challenges related to cell survival and immune rejection [37]. Myoblasts are fundamental for establishing the hierarchical structure that enables force transmission. Notably, C2C12, a murine satellite cell-derived, immortalized myoblast cell line, is commonly used in vitro to assess the impact of therapies or matrices on muscle regeneration. The fusion of myoblasts produces multinucleated myotubes, which further mature into myofibers, a functional unit of skeletal muscle. Myoblasts have been extensively studied in isolation and in conjunction with other cells, such as endothelial cells, to enhance vascularization [41]. Muscle progenitor cells (MPCs) have been explored for muscle regeneration in conjunction with scaffolds, but their xenogeneic source has limited their clinical application [28, 42].

Stem cells, including MSCs, perivascular stem cells, and adipose-derived stem cells, represent potential sources of myogenic cells. MSCs are easily isolated from various tissues and possess pluripotent characteristics and high proliferative potential, allowing differentiation into skeletal muscles, nerves, and blood vessels—essential components in skeletal muscle repair [43, 44]. Adipose tissues provide abundant adult pluripotent stem cells that can differentiate into adipogenic, chondrogenic, osteogenic, and myogenic cells [45]. These cells are easily isolated, expanded in vitro, and evade the immune system, minimizing the risk of host rejection [36, 46]. Studies have demonstrated that muscle-derived stem cells can enhance satellite cell seeding, support vessel formation, and reduce fibrotic tissue growth [47]. iPSCs, with similar properties as embryonic stem cells (ESCs), are readily producible, exhibit high proliferation rates in vitro, and can differentiate into multiple cell types [48, 49]. Despite overcoming ethical concerns associated with the use of ESCs, iPSCs raise issues related to generation costs, potential teratoma formation, batch-to-batch variation, immune rejection, lack of standardized controls, and regulatory barriers [50].

## Skeletal Muscle TE Strategies

**Fig. 3:**
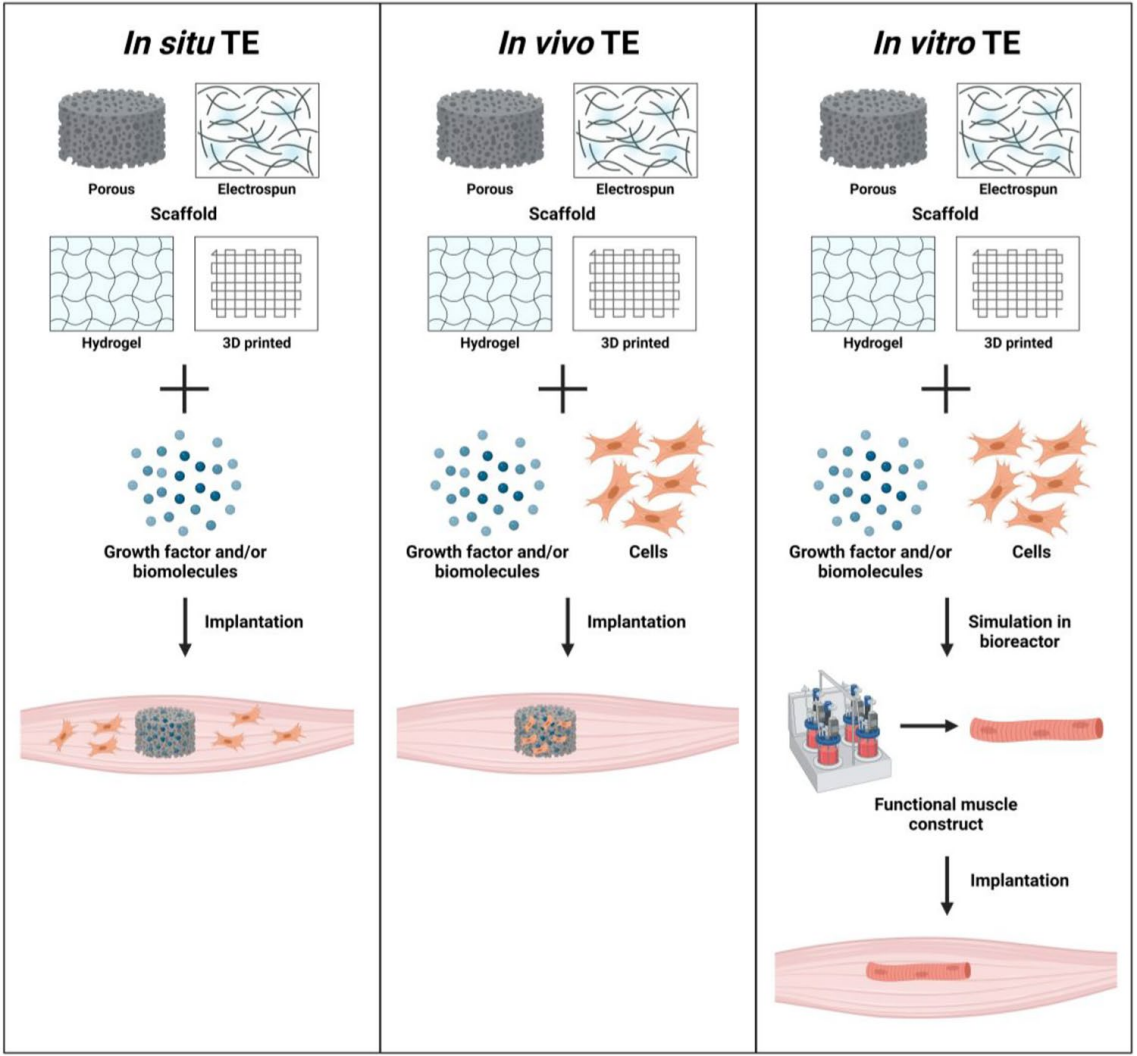
Different ways to engineer skeletal muscle using in situ, in vitro, and in vivo TE approaches. In situ TE relies on implanting an acellular scaffold, combining biophysical and/or biochemical cues, for stimulating endogenous tissue regeneration. In vivo TE transplants a cell-seeded scaffold and bioactive molecules for stimulating tissue regeneration. In vitro TE utilizes the implantation of a living, fully functional TE construct generated in vitro. Created with Biorender.com

Skeletal muscle TE can be broadly categorized into three main approaches: (i) in situ, (ii) in vivo, and (iii) in vitro TE (Fig. 3). These approaches differ in their incorporation of cells into the scaffold and the regeneration of skeletal muscle either in vitro vs. in vivo*.*

The in situ TE involves the implantation of acellular scaffolds to serve as templates for stimulating tissue regeneration in vivo. Strategically designed acellular scaffolds include biophysical and biochemical cues to recruit host cells, leading to their activation, proliferation, and differentiation at the injury site to regenerate skeletal muscle tissues, vasculature, and NMJs [51]. Acellular scaffolds offer easy and rapid fabrication, off-the-shelf availability, and long-term storage. Their lack of cells enables swift commercialization and approval by regulatory authorities like the Food and Drug Administration (FDA) [52]. However, conflicting results exist regarding the efficacy of this approach for treating VML injuries, primarily due to the restriction of endogenous satellite cells infiltrating the acellular scaffold within the defect [53].

The in vivo TE strategy involves transplanting a cell-seeded scaffold, where cells generate their local microenvironment to stimulate tissue regeneration in vivo. These scaffolds may incorporate bioactive molecules to enhance various cellular functions at the injury site. Tissue regeneration, vascularization, and NMJs are achieved by integrating with the host tissue and/or stimulating host’s response to promote new tissue formation. Initial attempts involved the direct delivery of cells into the defect through intramuscular injection but faced limitations regarding cell viability, retention, and susceptibility to host immune rejection [54]. Using scaffolds for cell delivery and cues often helps improve cell viability and engraftment. This strategy minimizes external manipulation of cells to preserve their functional properties, but the vulnerability of cells can lead to reduced viability, retention, and immune rejection [55, 56].

Lastly, in the in vitro TE approach, a living, fully functional TE construct is implanted. This is achieved by combining scaffolds, bioactive molecules, and cells, which are cultured in vitro until the cells differentiate into contractile myofibers along with the formation of blood vessels and NMJs [27]. Before implantation, these tissue constructs undergo pre-conditioning in a bioreactor with mechanical and electrical stimulation to stimulate differentiation and formation of functional muscle constructs. While this method yields constructs with higher functionality, it has drawbacks, such as the need for high cell density for cell differentiation, lower contractile forces compared to native tissues, and the inability to create complex structures [54]. Additionally, these constructs face limitations in size due to oxygen and nutrient requirements, requiring the formation of dense, complex vascular networks for the viability of metabolically active cells [28]. Accordingly, functional TE constructs are expensive and challenging to mass produce, maintain limited shelf availability, and face complex regulatory pathways for commercialization. Furthermore, significant variation in skeletal muscle tissue regeneration is observed due to variability in cell type, biomaterial, bioreactor conditions, growth factors, defect sizes, etc. [57].

## Scaffold Types for Skeletal Muscle Regeneration

### Decellularized ECM (dECM)

dECM is a prominent scaffold for addressing VML defects [58]. Derived from autogenic, allogenic, or xenogeneic sources, dECM scaffolds involve the removal of cells while preserving the intact 3D structure and chemical composition of the native ECM to guide tissue regeneration [30]. Rich in native ECM proteins, growth factors, and other essential biological molecules, dECM provides a conducive environment for the migration, proliferation, and differentiation of satellite cells into myotubes, which ultimately promotes the regeneration of functional skeletal muscle tissues [59]. Comprised of components such as collagen, fibronectin, laminin, elastin, heparin sulfate, HA, and proteoglycans, along with growth factors such as VEGF, FGF2, and TGF-β, dECM can be harvested from various source tissues, each contributing a unique 3D structure and chemical composition [60]. Commonly used tissues for dECM extraction include the dermis, pericardium, urinary bladder matrix (UBM), skeletal muscle, and small intestinal submucosa (SIS) [27]. One advantage of dECM scaffolds is their degradation by the host’s immune cells upon implantation at the injury site. Growth factors and other bioactive molecules are released during this process, which promotes the infiltration and activation of macrophages and the recruitment, proliferation, and differentiation of satellite cells and other progenitor cells [59, 61]. Studies indicate that ECM scaffolds enhance skeletal muscle tissue regeneration compared to the typical healing process, which often results in prolonged inflammation and scar tissue formation [52]. Furthermore, the degradation products from the ECM foster an M2-like macrophage phenotype, which stimulates the migration and differentiation of endogenous stem and progenitor cells and leads to the development of contractile tissue [62]. Consequently, these scaffolds are well-suited for VML injuries due to their ability to promote angiogenesis and the formation of new host ECM.

**Fig. 4:**
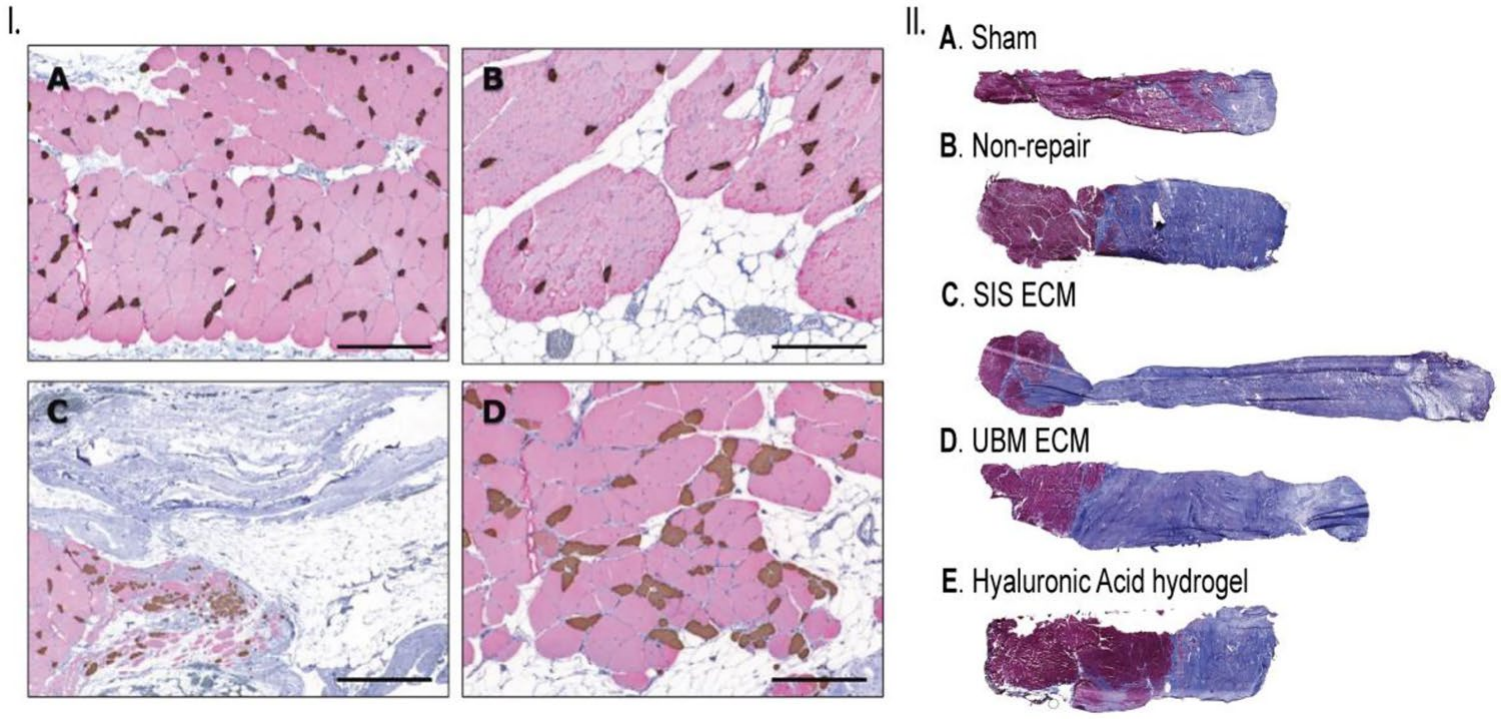
dECM-based scaffolds for skeletal muscle TE. **I** Porcine small intestinal submucosa (SIS)-based scaffold demonstrated the formation of skeletal muscle fibers in their native state, similar to native tissue. A: native tissue, B: SIS (uncrosslinked), C: SIS (crosslinked), and D: autologous tissue graft. Figure reprinted with permission from [66]. **II** Decellularized urinary bladder matrix scaffolds for repairing skeletal muscle injuries in animal model demonstrated extensive fibrosis in muscle after stained with Mason Trichrome’s (blue: connective tissue, red: skeletal muscle, and purple: nuclei). Figure reprinted with permission from [70]

Various tissues, including skeletal muscle, bladder, and small intestine, have served as sources of ECM for treating VML [63]. Implantation of SIS and muscle ECM scaffolds at the VML injury site has shown localized tissue formation, marked by cellular infiltration, myogenesis, vascularization, and innervated neuromuscular junctions after 6 months [64, 65]. Similarly, In a rodent abdominal wall model, Valetin et al. observed the development of functional skeletal muscle tissue at the VML injury site that exhibited morphology resembling native muscle, innervation, vascularization, and a composition of both type I and II muscle fibers (Fig. 4, I) [66]. Surprisingly, implantation of an ECM scaffold in a rodent tibialis anterior (TA) muscle VML model resulted in fibrosis at the implantation site [27, 67]. Despite the absence of new muscle fiber generation, improved functional outcomes were observed, possibly due to significant collagen deposition that provided structural support and prevented damage to the remaining muscle fibers [27, 67]. Garg et al. utilized muscle ECM scaffolds in a rodent TA model. Pro-inflammatory markers and a substantial macrophage content were observed around the implant 2 weeks post-implantation [68]. Although tissue formation initially took place in areas adjacent to the remaining tissues within 2 weeks, fibrous tissue was noted after 8 weeks, which contributed to improved functional outcomes, as previously reported [67]. In rodent and porcine VML injuries, UBM ECM scaffolds also demonstrated extensive fibrous tissue formation, limited muscle fiber generation, and persistent functional deficits (Fig. 4, II) [69, 70]. While these studies illustrate the potential of acellular scaffolds in regenerating skeletal muscle tissues, they also highlight the limitations of this approach in achieving de novo muscle tissue regeneration. This emphasizes the necessity for combinatorial approaches involving cells and bioactive molecules to achieve substantial muscle regeneration and functional recovery.

dECM is also the first TE scaffold used in humans. In 2010, a combat-injured marine with VML received an SIS ECM acellular scaffold 3.5 years post-injury, following an extensive physical therapy regimen [71]. After 4 months, the implant site showed new tissue formation, contributing to a modest improvement in isokinetic muscle performance [71]. Similarly, a 2014 study by Sicari et al. demonstrated comparable outcomes in patients with VML injuries (a minimum of 25% muscle mass loss and function) treated with a UBM ECM scaffold [52]. After 6 months, the authors documented the de novo development of muscle tissue at the implant site. Additionally, three patients exhibited a 20% improvement in the strength of the affected limb. These findings were supported by Dziki et al., who conducted a study involving 13 patients implanted with ECM scaffolds at the VML injury site [72]. Once again, the ECM scaffolds facilitated the formation of de novo, innervated, and vascularized tissue islands rich in perivascular stem cells, leading to a substantial increase in strength (37.3%) and range-of-motion (27.1%) 6 months postoperatively.

As dECM-derived scaffolds do not provide cells at the injury site, the success of skeletal muscle regeneration relies on endogenous cells to infiltrate the scaffold and create a dynamic microenvironment crucial for guiding the spatial and temporal phases of the regenerative process. Given the challenges related to cell infiltration in these acellular scaffolds, incorporating cells with biophysical and biochemical cues has proven instrumental in promoting muscle regeneration and significantly enhancing functional outcomes. For instance, Conconi et al. conducted a study comparing the performance of an acellular scaffold seeded with autologous myoblasts to non-seeded scaffolds implanted between the oblique abdominal muscles of rodents [73]. Thirty days post-implantation, the myoblast-seeded scaffolds exhibited numerous blood vessels and maintained their structural and functional integrity for an additional 30 days, while non-seeded scaffolds were encapsulated in scar tissue. Similarly, promising outcomes were observed when porcine UBM scaffolds, seeded with xenogeneic myoblasts, were implanted in nude mice with extensive VML injury [42]. Pre-conditioning of myoblast-seeded scaffolds in a bioreactor stimulated myotube alignment before implantation. Two months later, the cellular scaffold led to the formation of skeletal muscle tissue, evidenced by the presence of myofibers, blood vessels, and neurovascular bundles. The remodeled construct significantly improved force generation, with the maximal absolute isometric force reaching approximately 72% of that produced by the native muscle.

Mesenchymal stem cells (MSCs) are another frequently used cell type in skeletal muscle tissue regeneration. MSCs release growth factors in response to the microenvironment that modulate the immune system and direct the polarization of naïve macrophages toward the M2 phenotype—an essential process in skeletal muscle tissue regeneration [74]. Studies have demonstrated functional skeletal muscle regeneration after introduction of bone marrow-derived MSCs following crush injuries [75]. Qiu et al. explored the potential of porcine heart dECM and human umbilical cord MSCs, alone or in combination, for regenerating skeletal muscle tissue in rodent TA defects [76]. Muscle injuries treated with MSCs-seeded scaffolds exhibited the highest recorded isometric torque after 4 and 8 weeks of implantation compared to MSCs-alone and ECM-alone treatment groups. Additionally, the MSCs-seeded scaffold facilitated macrophage polarization toward the M2 phenotype while suppressing polarization toward the M1 phenotype, indicating scaffold remodeling. This regenerative microenvironment activated satellite cells that led to the formation of new muscle fibers. In another study, Merritt et al. implanted an ECM scaffold in a rodent VML defect and injected bone marrow-derived MSCs 7 days post-implantation [77]. Scaffolds injected with cells displayed significantly more blood vessel formation and myofibers than the ECM-alone treatment group, resulting in an impressive 85% functional recovery after 42 days. Further enhancement in functional recovery was observed with a higher cell count, revealing a direct correlation between the number of transplanted MSCs and functional muscle recovery [78].

In addition to direct seeding of cells within scaffolds, researchers have explored the delivery of minced muscle, i.e., skeletal muscle cut into small pieces, through ECM scaffolds. These minced muscle grafts contain resident elements crucial for de novo muscle fiber regeneration [79]. Kasukonis et al. used decellularized skeletal muscle as the scaffold to deliver minced muscle to a VML injury in a rodent model and reported significantly higher contractile force recovery and muscle mass restoration in groups treated with mince-muscle loaded scaffolds compared to untreated controls [80]. Goldman et al. employed a UBM ECM scaffold for delivering minced muscle to a rodent TA injury [81]. Although the treatment improved functional recovery with a 28.2% increase in peak isometric torque compared to untreated VML injury after 8 weeks, the observation of fibrous tissue deposition and interspersed islands of muscle fibers was noted.

Together, these studies showcase the diverse outcomes of dECM-derived scaffolds, when used independently and in conjunction with cells. This variability can be attributed to differences in ECM sources and variations in decellularization procedures, which influence the extent of cellular content removal, disruption of ECM structure, and damage to its biological activity [82]. The main limitations of dECM scaffolds include their inability to align incorporated or endogenously recruited cells and their high resorption rate in the body [58]. Pre-conditioning cellular scaffolds before implantation at the injury site has proven effective in enhancing the tetanic and twitch contractile responses of engineered muscle post-implantation in vivo [83]. Effective decellularization protocols are crucial to minimize disruption of the ECM’s 3D structure, architecture, and composition. An alternative avenue worth exploring is using cost-effective sources of ECM, such as various plants (e.g., broccoli, apple, carrot, sweet pepper), which exhibit physical similarities to human organs [84]. Despite these limitations, ECM remains the primary source material for regenerative constructs in VML treatment.

### Hydrogels, Sponges, and Meshes

Over the past few decades, hydrogels have found extensive applications in various biomedical applications, including skeletal muscle regeneration. These 3D crosslinked hydrophilic polymer networks can absorb significant amounts of water. Notably, hydrogels can effectively replicate the mechanical and structural characteristics of native tissues [85]. They can be synthetic or natural based on the biomaterial’s origin. Natural hydrogels are typically derived from collagen, elastin, chitosan, HA, alginate, and fibrin, while synthetic hydrogels are composed of synthetic polymers, such as PEG, PLA, PGA, PCL. Many hydrogels evaluated for skeletal muscle regeneration leverage natural polymers, including fibrin, collagen, chitosan, and HA [47, 86, 87]. Natural hydrogels offer the advantage of being cell adhesive and fostering robust cell interactions. Conversely, synthetic hydrogels exhibit lower biocompatibility and are associated with non-specific cell interactions [25].

As an alternative to dECM-based scaffolds, hydrogels offer the ability to finely adjust crucial properties, including porosity, degradation rate, and mechanical stiffness. Additionally, the incorporation of cells into hydrogels is often easily achievable through pre-polymerization mixing, allowing the formation of structures in various shapes. Hydrogels designed for muscle generation can either be pre-formed in vitro or injected at the injury site. Injectable hydrogels offer the advantage of administering the material directly to the injury site, conforming to the defect’s shape. In a study by DeQuach et al., an injectable hydrogel composed of collagen and dECM demonstrated promise in regenerating skeletal muscle in a rodent hindlimb ischemia model [88]. The injectable hydrogel promoted muscle progenitor cell proliferation and exhibited higher blood vessel density than collagen hydrogel alone. In another study, Marcinczyk et al. implanted a hydrogel consisting of fibrinogen and laminin-111 into a murine VML model [89]. Two weeks post-implantation, the hydrogel displayed increased infiltration of endothelial, hematopoietic, and immune cells. After an additional 2 weeks, the treated muscle exhibited induction of an anti-inflammatory M2-like macrophage phenotype and heightened myogenic activity, which indicated regeneration and functional recovery in VML-traumatized muscle. However, no significant increase in muscle weight and peak isometric torque was observed.

Hydrogels have been utilized to deliver various cell types, including myoblasts, MDPCs, MSCs, minced muscle, and adipose-derived stem cells [47, 90–94]. Beier et al. injected myoblasts suspended in a 3D fibrin matrix into a rodent muscle defect [95]. Seven days post-implantation, the authors observed the integration of injected myoblasts with host muscle fibers at the injection site, with no inflammatory response attributed to fibrin. Similarly, Rossi et al. utilized in situ photo-crosslinkable HA hydrogels to deliver satellite cells and MPCs in a murine TA model [96]. They found that satellite cells embedded in hydrogels demonstrated significant functional recovery, leading to the formation of new fibers with vascular and neural networks, compared to MPCs embedded in hydrogels or hydrogel alone. Hydrogels have also been explored for delivering minced muscle grafts to promote skeletal muscle tissue regeneration. Ward et al. delivered minced muscles using collagen hydrogels in a rodent VML model and observed that delivering 50% minced muscle resulted in functional improvements similar to 100% minced muscle grafts [91]. In another study, Goldman et al. demonstrated enhanced functional recovery, including the generation of new muscle fibers and improved repair of existing ones, achieved by delivering 50% minced muscle grafts through a laminin-111 supplemented HA hydrogel [92]. Additionally, the delivery of adipose-derived stem cells via collagen hydrogel enhanced myogenesis, angiogenesis, and polarization of macrophages toward the M2 phenotype that led to skeletal muscle tissue repair and regeneration 8 weeks post-injury [94].

Studies on muscle regeneration have investigated the use of thermosensitive, photosensitive, and magnetic hydrogels [87, 97, 98]. However, their suboptimal mechanical characteristics and lack of spatiotemporal cues impose significant limitations on their suitability for skeletal muscle regeneration. The successful engineering of the hydrogel microenvironment is recognized as a crucial factor in achieving functional muscle tissues. Techniques like 3D printing or photolithography integration allow the creation of spatial and temporal variations within the scaffold geometry, enabling precise regulation of a spectrum of cellular functions and downstream signaling pathways.

Previous research has emphasized the pivotal role of ECM stiffness in governing cellular function and influencing processes such as adhesion, spreading, proliferation, migration, and differentiation through mechanotransduction [99, 100]. MSCs exhibit distinct differentiation into various cell types based on substrate stiffness, with soft, stiff, and rigid matrices leading to neuronal, myoblastic, or osteoblastic differentiation, respectively [101]. Investigating the impact of hydrogel stiffness on muscle repair post-VML injury, Basurto et al. employed an HA-based hydrogel with tunable mechanical properties [87]. The hydrogels were designed with three stiffness levels: low (1 kPa), medium (3 kPa), and high (10 kPa), with high stiffness mirroring characteristics similar to healthy adult skeletal muscles, while low-to-medium stiffness emulated developing skeletal muscle microenvironments. Results indicated that HA hydrogels with medium stiffness significantly facilitated functional muscle recovery compared to hydrogels with low and high stiffness. The authors proposed that reduced functional recovery in high stiffness hydrogels may be attributed to a high crosslinking density limiting cellular infiltration within the hydrogels, consequently impeding scaffold degradation. This emphasizes the need for optimal hydrogel stiffness and pore size to promote adequate cellular infiltration and scaffold remodeling, which is crucial for regulating cellular functions. Boontheekul et al. further demonstrated how the combination of cell type, mechanical stiffness, and degradation rate can influence the myoblast’s phenotype and differentiation [31]. Although alginate-based hydrogels are biocompatible, biodegradable, and non-immunogenic, they exhibit weak cell adhesion, necessitating functionalization with adhesive ligands such as integrin and RGD. Ansari et al. developed RGD-coupled alginate hydrogels with varying stiffness to elucidate their role in cell viability and myogenic differentiation of encapsulated gingival MSCs [102]. They found that MSCs encapsulated in hydrogels with intermediate stiffness (10–16 kPa) demonstrated the highest myogenic differentiation compared to hydrogels with low (< 5 kPa) and high stiffness (> 20 kPa). Moreover, implantation of MSCs encapsulated in alginate hydrogel, supplemented with a myogenic differentiation cocktail, resulted in the formation of muscle-like tissue with improved vasculature compared to bone marrow MSCs encapsulated in alginate hydrogel.

**Fig. 5:**
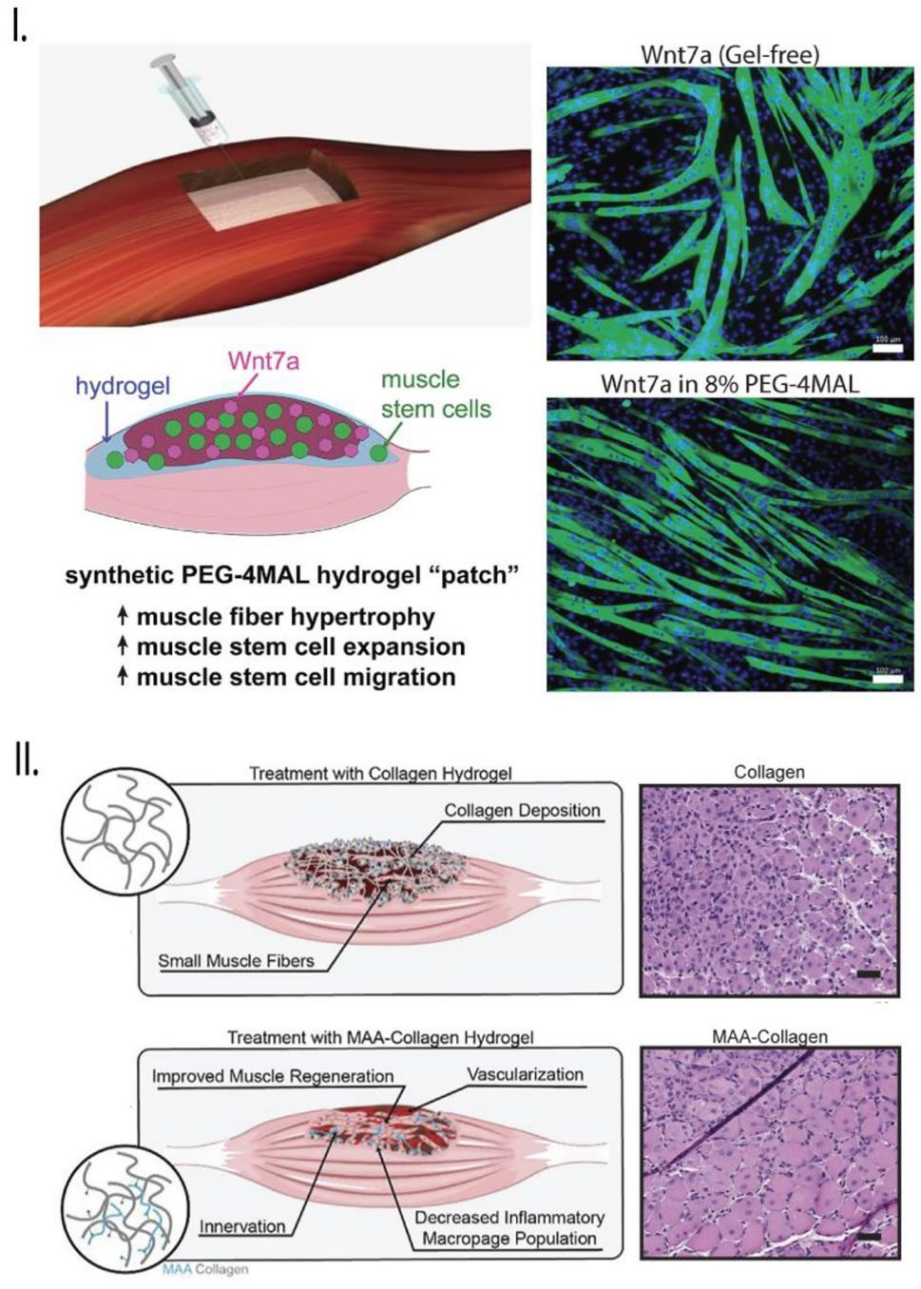
Hydrogels as scaffolds for promoting muscle regeneration. **I** A Wnt7a-loaded injectable and bioadhesive PEG-maleimide hydrogel, when treated with C2C12 cells, led to highly aligned myotubes due to effective controlled delivery of Wnt7a in-vitro. Figure modified and reprinted with permission from [103]. **II** A methacrylic acid-collagen based hydrogel, when implanted at the site of VML promoted muscle regeneration with enhanced vascularization and phenotypic switching of macrophages into an anti-inflammatory M2 state. The hydrogel exhibited enhanced diameter of the centronucleated Muscle Fibers (MFs) compared to other polymeric systems. Figure modified and reprinted with permission from [105]

In addition to natural hydrogels, synthetic hydrogels have been used as scaffolds for skeletal muscle regeneration, offering a key advantage in easily customizing their mechanical and structural properties according to the requirements of TE applications. For instance, Han et al. developed a synthetic polyethylene glycol (PEG)-based hydrogel tailored to deliver muscle satellite cells and Wnt7a, a pro-myogenic factor, and investigated its potential in promoting skeletal muscle regeneration following a freeze-injured TA muscle (Fig. 5, I) [103]. Results demonstrated that the co-delivery of satellite cells and Wnt7a led to increased muscle fiber hypertrophy and enhanced cellular migration during the engraftment process, significantly improving muscle regeneration after 2 weeks compared to Wnt7a-free hydrogels. In another study, Salimath et al. identified various parameters, such as polymer weight percentage, functionalization with adhesive ligands, and growth and differentiation media, to successfully differentiate MPCs into functional, contractile muscle tissues [104]. The combination hydrogel of methacrylic acid (MA) with PEG and collagen was also tested for regenerating skeletal muscle tissue in a murine VML injury model (Fig. 5, II) [105]. While both hydrogels exhibited enhanced vascular networks, only MA-collagen hydrogels demonstrated muscle innervation that resulted in improved muscle regeneration characterized by increased muscle fiber size and force production. Recombinantly expressed proteins have also been harnessed for skeletal muscle TE applications. Due to their recombinant nature, these proteins can be easily modified to incorporate functional biological sites by altering their DNA sequence through cloning and site-directed mutagenesis techniques. An example is the elastin-like protein (ELP) for repairing skeletal muscle following a VML injury [106]. These recombinant ELP-based hydrogels significantly enhanced skeletal muscle regeneration by modulating the macrophage phenotype, preventing fibrosis, and forming myofibers with a morphology similar to that of healthy muscle.

Sponges and meshes represent another category of scaffolds designed to provide a porous structure for supporting cell viability and facilitating migration. Creating a sponge-based scaffold involves the straightforward process of freeze-drying the polymer solution, resulting in a porous structure with variable pore sizes. An alternative technique for crafting porous PLGA scaffolds is gas foaming, which employs high-pressure gas without organic solvents and high temperatures [107]. After implantation into a muscle defect, collagen sponges demonstrated a reduction in scar tissue formation and a more significant presence of regenerating myofibers, including increased number, diameter, and length, compared to untreated muscle [108]. Haas et al. developed biomimetic sponges made of collagen, gelatin, and laminin, crosslinked with 1-ethyl-3-(3-dimethyl aminopropyl) carbodiimide [109]. Two weeks post-implantation in a murine VML model, the crosslinked sponges exhibited satellite cell infiltration, endothelial cell presence, and inflammatory cell infiltration, which fostered myofiber regeneration at the injury site. Utilizing PGA meshes, Saxena et al. delivered myoblasts for skeletal muscle regeneration [110, 111]. After 6 weeks, the degradable PGA meshes supported cell viability to enable myoblast organization and the regeneration of new tissue, accompanied by developing a vascular network. Similar outcomes were observed by Kamelger et al., who tested three different biomaterials-PGA meshes, alginate, and hyaluronic acid (HA) hydrogels-to deliver cells and investigate their potential for regenerating skeletal muscle within a pre-formed capsule in a rodent model [112]. Although no cell migration into the surrounding tissue was noted, all three biomaterials formed myotubes within the capsule, highlighting their potential in skeletal muscle TE.

### Aligned Scaffolds

Skeletal muscle is composed of myofibers that organize into fascicles and larger bundles. This arrangement results in an anisotropic structure of the ECM, which aligns with the muscle axis to facilitate force generation during muscle contraction [113]. Cell alignment is a pivotal factor in skeletal muscle growth [114]. Various techniques, such as microfabrication, electrospinning, microthreads, and aligned pores, have been used to establish anisotropic surface topography.

Huang et al. used microfabrication techniques to create micropatterned poly(dimethylsiloxane) (PDMS) microgrooves that resulted in improved alignment and fusion of myoblasts along these microgrooves compared to non-patterned surfaces [115]. Moreover, the organized cellular alignment persisted when these aligned myotubes were transferred into collagen hydrogels, forming well-aligned tissue-engineered muscle constructs. Similarly, Lam et al. created engineered muscles by integrating aligned myotubes into fibrin hydrogels with enhanced muscle fiber content and increased force generation [116]. Charest et al. investigated the influence of diverse topographies on myoblast alignment and differentiation, such as ridges/grooves and holes arranged in an orthogonal array [117]. These geometries did not impact cell density and differentiation, even though they influenced cell alignment. Conversely, Wang et al. highlighted the significance of groove depth over groove width, showing its greater influence on cell morphology, proliferation, and differentiation in skeletal muscle regeneration [118]. In a comprehensive study, Huang et al. combined nanoscale and microscale topographical cues to successfully induce alignment in myoblasts and cytoskeletal proteins and promote myotube assembly and striation [119]. Notably, nanoscale features effectively suppressed myoblast proliferation during differentiation and fusion.

**Fig. 6:**
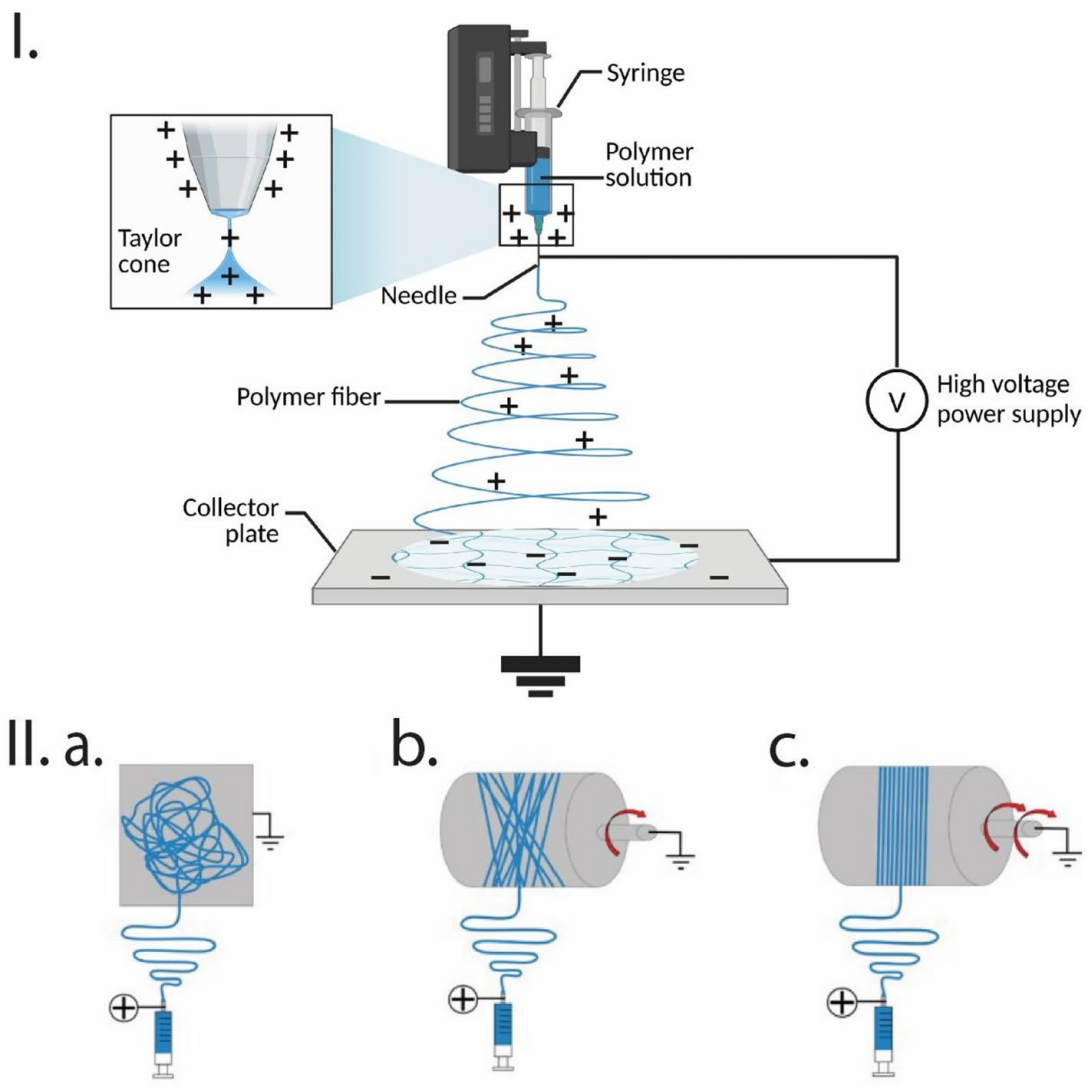
Overview of electrospinning for generating random and aligned nanofibers. **I**. During electrospinning, a high voltage is applied to a liquid droplet formed by the polymer solution, which results in the elongation of the droplet at the tip of the needle because of the overpowering of the surface tension by the large repulsive force. A “Taylor cone” is formed at a threshold voltage, leading to liquid discharge from the needle that eventually gets deposited on the collector in the form of fibers. **II** Generation of randomly aligned fibers using (a) a flat plate collector or (b) a drum collector rotating at low speeds. (c) Highly aligned fibers are obtained when the drum collector is rotating at high speeds. Created in Biorender.com and reprinted with permission from [121, 122]

Electrospinning is a well-established technique renowned for producing nanofibrous scaffolds with a substantial surface-area-to-volume ratio and high porosity. These features make them well-suited to mimic the nanoscale characteristics of the native ECM. Electrospinning can produce aligned nanofiber scaffolds that promote cell adhesion, proliferation, and cellular alignment, and are therefore ideal candidates for skeletal muscle TE [27]. Electrospun scaffolds can be tailored with anisotropic and isotropic orientation, variable thickness, and nanoscale pores to modulate cellular behavior [120]. Figure 6 illustrates the electrospinning setup to generate nanofibers with random and aligned orientation [121, 122]. A variety of synthetic and natural materials are amenable to electrospinning. Moreover, it enables the creation of scaffolds with diverse morphologies, fiber alignment, fiber diameters, and porosity [123].

Nakayama et al. engineered 3D parallel-aligned collagen nanofibrillar scaffolds to address VML injury in a murine TA model coupled with rehabilitative exercise [124]. VML-injured muscles treated with these aligned collagen scaffolds exhibited a significantly higher myofiber cross-sectional area than those treated with decellularized scaffolds or left untreated. Additionally, a notably higher density of perfused microvessels was observed compared to treatments involving randomly oriented nanofibers. In a separate study, endothelial cells and myoblasts were cultured within these aligned nanofibrillar collagen scaffolds. This resulted in the organized assembly of longer myotubes, enhanced contractility, and the development of a microvascular network, which surpassed the outcomes seen in muscles formed from cells seeded on randomly oriented scaffolds [41]. The creation of highly aligned myofibers and a well-established vascular network led to superior vascular perfusion compared to treatment with randomly oriented scaffolds. The combination of collagen and PCL was utilized to create nanofiber meshes with different orientations [125]. Only cells seeded onto highly aligned scaffolds induced cell alignment and myotube formation, unlike cells seeded on randomly oriented fibers. In a rat VML model, myoblasts and motor neurons were co-cultured on PCL fibers prior to implantation. The pre-innervated tissue-engineered muscle construct exhibited an increased density of satellite cells, enhanced vascularization, and improved innervation at the implantation site and led to an increase in muscle cross-sectional area [58]. Beldjilali-Labro et al. combined microfabrication and electrospinning to generate PCL nanofibers coated with Ag nanoparticles that were further micropatterned with PEG hydrogel lines [126]. These scaffolds increased myoblastic differentiation, which formed dense and aligned myotubes that eventually fused to create larger myofibers.

Fibrin is another fibrillar protein employed in skeletal muscle regeneration. Guo et al. used the solution-electrospinning method to encapsulate myoblasts into fibrin-polyethylene oxide (PEO) hydrogel microfibers [127]. Within a week, the encapsulated myoblasts exhibited significant proliferation, elongation, and differentiation within the fibrin microfiber bundles. Similarly, Gilbert-Hornick et al. seeded adipose-derived stem cells on uniaxially aligned fibrin microfibers [128]. Although the seeded cells exhibited alignment and elongation, they did not fully recapitulate myotube characteristics even after 2 months of in vitro culture. Nevertheless, when implanted in a VML defect model, the cell-seeded fibrin fibers contributed to skeletal muscle regeneration by integrating with the host tissue and promoting cellular and vascular growth with minimal fibrosis formation. Likewise, myoblasts seeded onto electrospun fibrin scaffolds exhibited substantial skeletal muscle regeneration with high myofiber density and a well-established vascular network compared to acellular scaffolds lacking muscle regeneration 2 and 4 weeks after implantation [129]. The fibrin scaffold completely degraded after 2 weeks in vivo and was replaced with regenerating muscle tissue.

The application of dECM has expanded to creating aligned fibers for skeletal muscle TE. Smoak et al. successfully fabricated dECM-based electrospun scaffolds without a carrier polymer and tailored their physiochemical properties to mimic those of native muscle tissues [130]. These highly porous scaffolds facilitated nutrient transport and metabolic waste removal, and the addition of a crosslinking agent enhanced their tensile modulus. In another approach, Patel et al. developed a composite nanofiber consisting of dECM and PCL to improve the mechanical properties of the dECM scaffold and evaluated myogenesis in vitro [131]. Primary satellite cells cultured on anisotropic PCL-dECM and PCL scaffolds displayed alignment along the nanofiber direction, contrasting with cells on isotropic scaffolds, which exhibited disorganization with a random distribution, likely attributable to the random arrangement of fibers. The PCL-dECM nanofibers demonstrated higher cell proliferation than PCL nanofibers alone, emphasizing enhanced differentiation capabilities compared to the PCL scaffold alone. The authors proposed that the low substrate stiffness and pre-regenerative proteins on the PCL-dECM scaffold promoted satellite cell proliferation while suppressing their differentiation. When implanted in a murine VML model, the presence of anti-inflammatory M2 macrophages and myofiber regeneration was increased on these scaffolds compared to no treatment or the PCL scaffold alone [10]. However, no significant functional improvement was observed in muscle weight and force production. Smoak et al. fabricated skeletal muscle dECM-based electrospun fibers with increasing fiber alignment and crosslinking density, which correlated to the mechanical strength of the scaffold [132]. Increased crosslinking density was found to reduce cell adhesion and proliferation, thereby impacting myotube formation. This indicates that the potential of dECM-based electrospun fibers can be enhanced by tuning these properties.

Wang et al. introduced a novel approach by developing monodispersed magnetically controlled short nanofibers (MSNF) encapsulated within a photocurable methacrylated gelatin hydrogel, alongside myoblasts, using a magnetic field [97]. This method produced an anisotropic, aligned microstructure that, similar to other aligned scaffolds, induced myoblasts to attach, spread, and differentiate into aligned myotubes. The scaffold was injected into a rodent TA VML model, and a magnetic field was applied to align MSNFs along the direction of muscle contraction. The added magnetic field promoted the formation of newly aligned myofibers, larger in diameter, to contribute to enhanced functional recovery in VML-injured muscles.

Microthreads offer a distinct structural component that can be assembled and crosslinked into tissue-specific structures with precisely engineered morphological, mechanical, and biochemical cues to support scaffold-guided tissue remodeling [133, 134]. Crafted from fibrous materials like collagen, fibrin, silk, and alginate, microthreads can be combined to form various structures, including bundles, aligned sheets, or braided scaffolds. Cornwell et al. produced fibrin microthreads by combining fibrin and thrombin with UV crosslinking to enhance scaffold strength and stiffness [135]. However, following UV crosslinking, cell–matrix interactions were altered and fibroblast proliferation was reduced compared to uncrosslinked fibrin microthreads. Similarly, Page et al. developed cell-loaded fibrin microthreads to enhance skeletal muscle regeneration [136]. Cell-seeded fibrin microthreads, when implanted in a murine TA model, facilitated muscle fiber formation, reduced collagen deposition and supported stable engraftment, which led to approximately 100% functional recovery after 3 months. Crosslinking density impacted the mechanical, structural, and biochemical properties, which ultimately influencing cellular behavior [133]. Additionally, techniques like unidirectional freezing can produce collagen sponges with aligned pores, which also promote aligned myotube formation in vitro and muscle fiber formation in situ upon implantation in a murine TA model [137]. Similarly, Jana et al. created uniaxially aligned porous structures of chitosan by varying polymer concentration and applying temperature gradient during freezing [138]. These chitosan scaffolds supported myoblast adhesion, proliferation, and differentiation into myotubes, with the myotube diameter proportional to the scaffold’s stiffness.

### Electromechanically Preconditioned Scaffolds

Skeletal muscle tissue is comprised of multinucleated myofibers responsible for generating movement forces, and its functionality heavily depends on electrochemical stimulation from motor neurons. This stimulation plays a crucial role in myoblast differentiation and the maintenance of mature myofiber function. While scaffolds provide biophysical and biochemical cues, emulating in vivo stimuli through mechanical and electrical stimulation becomes essential for activating cell signaling pathways pivotal in skeletal muscle tissue development [139]. Mechanical stimulation emulates the uniaxial strains experienced during daily movements, while electrical stimulation induces muscle contraction, mirroring motor neuron electrical synapses. Research indicates that a lack of both mechanical (resulting from immobility) and electrical (due to denervation) stimulation leads to muscle atrophy, whereas their application enhances the maturation and contractility of skeletal muscle tissue [140]. Interestingly, the combined application of electrical and mechanical stimulation proves particularly effective in promoting the maturation and contractility of skeletal muscle tissue [141]. In skeletal muscle TE, electromechanical stimulation has proven instrumental in promoting myoblast fusion, alignment, hypertrophy, and contractility during in vitro muscle engineering [83, 142].

Conductive biomaterials are pivotal in facilitating electromechanical stimulation within the field of skeletal muscle TE. Commonly utilized materials include polyaniline (PANi), polythiophene, polypyrrole (PPy), carbon-based nanotubes, graphene, gold (Au), and silver (Ag) nanoparticles. Each of these materials offers distinct advantages and disadvantages regarding biodegradability, conductivity, processability, water solubility, and immunogenicity [35]. Consequently, these conductive biomaterials are frequently integrated with natural or synthetic biomaterials to optimize their applications in TE. Basurto et al., for instance, developed aligned and electrically conductive collagen and chondroitin sulfate scaffolds by incorporating PPy [141]. This approach enhanced myotube formation, maturation, and an organized cytoskeleton structure along the collagen backbone, reminiscent of healthy skeletal muscle. Electrically conductive PCL-graphene nanocomposites, supplemented with extracellular zinc ions as myogenic factors, proved biocompatible and promoted increased proliferation and differentiation of seeded myoblasts compared to non-conductive scaffolds [143].

Based on these initial findings, Basturkmen et al. developed Ag-nanowire-loaded PCL composite nanofiber scaffolds to combine the topographical cues and conductive microenvironment for regenerating skeletal muscles [144]. Myoblasts seeded on these composite nanofibers thrived irrespective of electrical stimulation. This was further supported by Jeong et al., who demonstrated that myoblasts cultured on conductive PANi-PCL nanofibers displayed superior cell adhesion compared to their counterparts on non-conductive PCL nanofibers [145]. Chen et al. further illustrated that electrically conductive and aligned PANi/PCL scaffolds significantly enhanced myoblast orientation, promoted the formation of myotubes, and facilitated their maturation [146]. When satellite cells were cultured on electroconductive PANi/polyacrylonitrile scaffolds, robust proliferation occurred on soft surfaces, while enhanced differentiation was observed on stiffer surfaces [147]. Velasco-Mallorquí et al. generated a functional TE skeletal muscle using a gelatin-cellulose scaffold doped with carbon nanotubes [148]. This composite scaffold, characterized by a highly aligned morphology, not only supported viability, cell alignment, and cell fusion but also facilitated myotube formation by the encapsulated myoblasts. The application of electrical stimulation proved crucial in promoting myotube maturation, a phenomenon consistently observed in vivo when electrically stimulated scaffolds were implanted. Serena et al. employed collagen scaffolds seeded with MDPCs and provided periodic electrical stimulation beginning 3 days after cell seeding [142]. After 7 days of in vitro maturation, MDPCs exhibited elevated levels of myogenic markers compared to unstimulated cells. Implantation of the stimulated scaffold into the TA muscle of mice led to new fiber formation 10 days post-implantation. Researchers have also explored electroconductive hydrogels. Sasaki et al. fabricated conductive double-network hydrogels based on poly (3,4-ethylenedioxythiophene) (PEDOT) and polyurethane and demonstrated increased cell adhesion and viability compared to non-conductive double-network hydrogels [149]. In a separate study, conductive gelatin methacryloyl (GelMA) hydrogels embedded with palladium-based metallic glass sub-micron wires enhanced cell adhesion, spreading, elongation, and myoblast differentiation into myotubes when compared to GelMA hydrogels alone [150]. The application of electrical stimulation further enhanced the contraction behavior and metabolic activity of myotubes within these hybrid hydrogels.

Like electrical stimulation, cyclical mechanical conditioning has emerged as a powerful catalyst for myotube formation and maturation. Grossi et al. studied the impact of mechanical stimulation in conjunction with specific integrin receptors, specifically fibronectin and laminin, on myoblast proliferation and differentiation [151]. Myoblasts stimulated through laminin receptors, both chemically and mechanically, exhibited enhanced proliferation and differentiation compared to those stimulated via fibronectin receptors. The application of mechanical forces further enhanced differentiation and fusion. In another study, Moon et al. seeded MPCs onto dECM scaffolds and subjected them to uniaxial strain to examine how cell-seeded scaffolds respond to cyclic mechanical stimulation [83]. Pre-conditioning constructs with a bioreactor produced viable skeletal muscle tissue constructs with cellular alignment, organization, and maturation within 5 days. In contrast, constructs that were not pre-conditioned lacked cellular and tissue organization. After 3 weeks, these constructs demonstrated contractile responses and generated a tetanic response when implanted subcutaneously into mice. Notably, pre-conditioned constructs exhibited cellular alignment, tissue organization, and a tetanic contractile response, reaching 1% of native tissue levels, whereas without pre-conditioning, constructs showed no tetanic response 4 weeks post-implantation. Similar results were obtained when myoblasts seeded on UBM dECM were implanted in murine VML defect after a week of pre-conditioning [42]. Two months post-implantation, pre-conditioned constructs exhibited remodeling with the presence of myofibers, blood vessels, and NMJs. In addition, the mechanically pre-conditioned constructs generated a maximum tetanic force of ≈ 72% of the native muscle, while the non-treated or implanted with acellular dECM produced only ≈ 50%. Similar success was replicated by Passipieri et al., where bladder acellular matrix seeded with MPCs, pre-conditioned with uniaxial mechanical strain, demonstrated significant de novo muscle regeneration with approximately 90% functional recovery in a rat VML injury defect [152]. Furthermore, the combination of an extended cellular maturation period in the presence of bioreactor conditioning, coupled with a secondary application of MDCs onto the layer of maturing cells, led to rapid and long functional recovery and produced a twofold increase in force production compared to other tested constructs [153]. However, the implantation of TE muscle constructs following mechanical stimulation in rodent TA defects yielded variable responses in functional skeletal muscle regeneration, possibly due to differential immune responses [154, 155].

Static mechanical strain has also proven helpful in developing TE muscle constructs. Gholobova et al. co-cultured MDPCs and endothelial cells in a fibrin hydrogel under tension and identified the optimal parameters, such as such as cell density, cell population ratios, and culture media, to obtain densely packed aligned myofibers with an interspersed vascular network [156]. Similarly, Juhas et al. developed biomimetic skeletal muscle tissues by casting a Matrigel/fibrin matrix seeded with myoblasts into PDMS molds and applying uniaxial tension at both ends [157]. Upon in vivo implantation, these TE constructs exhibited highly aligned myofibers and a robust vascular network, and elicited strong twitch contractions upon electrical stimulation.

The synergistic application of electrical and mechanical stimulation has improved myoblast differentiation into mature myotubes and enhanced functional recovery. Liao et al. engineered highly aligned PU fibers with variable stiffness and investigated their impact on myotube formation and maturation [158]. Myoblasts cultured on aligned PU fibers displayed enhanced cell elongation and alignment, and improved cytoskeleton organization. The study emphasized the crucial timing of electromechanical stimulation in modulating myoblast differentiation, specifically by applying electrical stimulation after myotube assembly and mechanical stimulation before myoblast differentiation. Synchronized electromechanical stimulation significantly increased the percentage of striated myotubes, upregulated the production of contractile proteins, and presented a more efficient approach to achieving a mature TE muscle construct.

### Scaffolds Incorporating Growth Factors and Other Bioactive Molecules

As discussed earlier, integrating both biophysical and biochemical cues into scaffolds proves instrumental in expediting scaffold remodeling and facilitating the regeneration of injured skeletal muscles. To optimize the therapeutic efficacy of delivering cells via a scaffold, growth factors can be co-administered with cell populations at the designated injury site. In the context of skeletal muscle regeneration, various growth factors, such as basic fibroblast growth factor (bFGF), insulin-like growth factor (IGF), platelet-derived growth factor-B (PDGF-B), transforming growth factor beta (TGFβ), hepatocyte growth factor (HGF), vascular endothelial growth factor (VEGF), and others play crucial roles in sustaining the proliferation and differentiation of satellite cells (Table 2) [159]. The incorporation of these growth factors within scaffolds enhances the potential of ECM by promoting cell survival and activating downstream cell signaling pathways for muscle regeneration. Additionally, the control over release kinetics, localized delivery, and therapeutic concentration of these growth factors can be achieved by adjusting the scaffold’s crosslinking density, porosity, and degradation rate.

**Table 2:** Role of commonly utilized growth factors in the development of skeletal muscle tissue constructs

Growth factor	Role in skeletal muscle regeneration
HGF	Activates quiescent satellite cells and their migration to the injury site [160] Facilitates angiogenesis in combination with VEGF [161] Promotes satellite cell proliferation [162]
bFGF	Promotes satellite cell proliferation and differentiation [162] Promotes neurogenesis [163] Promotes endothelial cell proliferation and migration [164]
IGF-1	Promotes satellite cell proliferation and differentiation and increases force production [165] Stimulates myoblast proliferation and differentiation and myotube formation [166–168] Reduces fibrosis and inflammation [169]
VEGF	Increases neovascularization and myofiber regeneration [170] Promotes satellite cells activation and proliferation, and myoblast migration and survival [171] Promotes endothelial cell migration and proliferation [172]
TGF-β	Inhibits satellite cell proliferation and differentiation and promotes fibroblast proliferation; increases contractility [173–175]
PDGF-B	Promotes myoblast proliferation and myotube maturation [176]

Numerous studies have investigated the impact of growth factor delivery on the survival, proliferation, and differentiation of encapsulated cells. In a study by Hagiwara et al., GFP+-myoblasts were transplanted with gelatin microspheres containing bFGF into a rat’s injured thigh muscle [177]. Four weeks post-transplantation, muscles treated with the combination of myoblasts and bFGF exhibited a significantly higher survival rate of transplanted myoblasts compared to myoblasts alone or with empty microspheres. The controlled and sustained release of bFGF further promoted myoblast differentiation and enhanced overall muscle regeneration. However, Hu et al. did not observe significant therapeutic effects of bFGF-laden collagen nanofibrillar scaffolds compared to bFGF or collagen alone along among treatment groups [178]. Alcazar et al. investigated the synergistic effects of spatial patterning cues and IGF-1 delivery in conjunction with voluntary exercise after a VML injury in mice [168]. After implantation in the TA muscle, anisotropic, IGF-1-loaded, collagen nanofibrillar scaffolds enhanced myogenesis, prevented fibrous tissue formation, and significantly increased the density of the vascular network and mature NMJs compared to the same treatment without exercise. In another study, Grasman et al. implanted HGF-loaded, crosslinked, fibrin microthread scaffolds in a mouse VML model [179]. HGF release at the implantation site enhanced the differentiation of myoblasts and promoted angiogenesis. De novo myofibers formed that were aligned with healthy tissue. This combination treatment reduced the size of the injury and significantly improved functional recovery with a generated force approximately 200% higher than that of injured muscles. The delivery of VEGF at the injury site has also demonstrated the potential to promote neural functions and innervation in damaged tissues. Shvartsman et al. delivered VEGF using alginate hydrogel, while Lee et al. incorporated VEGF-laden PLGA microspheres within alginate hydrogels to prolong the release of the growth factor [180, 181]. In both studies, VEGF release promoted new blood vessel formation and revascularization at the implantation site. Shvartsman et al. also observed a reduction in axon degradation and the promotion of NMJ remodeling, highlighting VEGF as a facilitator of crosstalk between vascular and neural networks [181, 182].

Several studies have explored the therapeutic potential of delivering multiple growth factors in a spatiotemporal pattern to emulate the natural microenvironment of tissue formation, repair, and remodeling to improve regenerating outcomes. A frequently explored combination for promoting skeletal muscle regeneration is VEGF and IGF-1 [170]. After 6 weeks, injured TA muscles treated with cells, VEGF, and IGF-1 delivered via an alginate scaffold exhibited reduced fibrotic tissue formation, enhanced engraftment and vascularization at the implantation site, and significantly recovered contractile function compared to other experimental conditions. Wang et al. achieved a 90% recovery of tetanic contractile forces by delivering VEGF/IGF-1 and myoblasts through shape-memory alginate scaffolds [183]. In another study, an alginate scaffold containing MSCs, VEGF, and IGF-1 demonstrated sustained release and stimulated MSCs to secrete increased paracrine factors that regulated MDPC’s function and promoted survival, proliferation, and migration while delaying differentiation [184]. Co-delivery of MSCs and VEGF/IGF-1 in a clinically relevant crush injury model resulted in significant tissue remodeling, increased myofiber density, and enhanced functional recovery compared to other treatments. In a rat TA VML injury model, co-delivery of IGF-1, bFGF, and MDPCs using keratin scaffolds led to the formation of new myofibers and blood vessels and contributed to functional recovery [185]. However, statistical significance was not observed when injured muscles were treated with keratin hydrogels alone or containing growth factors. Baker et al. reported enhanced recovery of contractility using acellular keratin scaffolds containing bFGF and IGF-1 compared to similar scaffolds containing MDPCs [186]. Hill et al. explored a different growth factor combination, FGF2 and HGF, to promote skeletal muscle regeneration in a mouse TA injury model [187]. Alginate scaffolds containing myoblasts and growth factors promoted higher cell engraftment and muscle regeneration, which resulted in significant mass muscle recovery and complete resolution of the injury defect 30 days post-implantation. These studies highlight the stimulatory role of growth factors in regenerating skeletal muscle tissues. However, conflicting data necessitates further investigation into biomaterial selection, cell type, strategies for incorporating cells and growth factors, and electromechanical stimulation to maximize the regenerative potential of growth factor-loaded scaffolds.

In addition to growth factors, researchers have explored the delivery of other bioactive molecules, such as lipids, to manipulate the pro-inflammatory phase in skeletal muscle regeneration [188]. San Emeterio et al. engineered PLGA/PCL nanofibrous scaffolds loaded with Fingolimod (FTY720) to modulate Sphingosine-1-phosphate (S1P)-associated signaling pathways. This approach aimed to address the dysregulated immune microenvironment characteristic of VML injuries, often leading to chronic inflammation and pro-fibrotic signaling. The murine VML injury model study demonstrated that FTY720 delivery promoted the infiltration of pre-regenerative immune cells and activated MuSCs at the implantation site by regulating S1P receptors and associated signaling pathways. This alteration in the immune microenvironment facilitated the formation of aligned myofibers with larger diameters, enhanced revascularization, and improved reinnervation compared to untreated VML injuries. Pandolfi et al. utilized a chitosan-gelatin scaffold functionalized with PLGA-multistage vectors (MSV) to achieve the controlled and sustained release of S1P from PLGA microspheres at the target site [189]. S1P, a platelet-derived lipid mediator, regulates cell–cell and cell–matrix adhesion and enhances cell migration, differentiation, proliferation, and survival [190]. The composite scaffold maintained a sustained long-term release of S1P for up to 10 days, contrasting with PLGA microspheres, where 80% of the molecule was released within 48 h. Another approach by Haas et al. involved developing biomimetic sponges based on collagen, gelatin, and laminin-111 for the delivery of FK-506, an FDA-approved immunosuppressant drug, to modulate the immune response in a rat VML injury model [191, 192]. The biomimetic sponge significantly enhanced myofiber regeneration, reduced fibrosis, and improved torque production in injured muscles. These findings underscore the importance of identifying new biomarkers and signaling pathways crucial in skeletal muscle injury, repair, and remodeling.

## Limitations

In conclusion, VML poses a significant clinical challenge due to the scarcity of effective treatments capable of fully restoring functional recovery in affected patients. Despite efforts to develop regenerative therapies aimed at repairing injured muscles, promoting tissue regeneration, and restoring function, the outcomes have often fallen short in terms of volume, strength, and overall function of the muscle. VML injuries trigger robust fibrosis and prolonged inflammation, potentially leading to alterations in associated signaling pathways [193]. These changes in the microenvironment, encompassing structural, biochemical, and biomechanical cues, establish a detrimental feedback loop that impedes myogenic repair [194]. The delivery of biochemical and biophysical cues via scaffolds within this compromised environment limits their potential for tissue regeneration. Initial approaches to address VML could involve establishing a more conducive microenvironment for regenerative therapies to operate effectively. This may include the use of immunomodulatory and/or anti-fibrotic agents.

Another major challenge limiting the translation of TE constructs into clinical trials is the necessity for their in vivo evaluation in appropriate animal models. While rats and mice remain the predominant preclinical animal models for assessing the regenerative potential of TE constructs in skeletal muscles, it’s crucial to acknowledge the considerable variation in dimensions of VML defects created in these animals, ranging from partial-thickness to full-thickness injuries. For instance, in the case of mouse quadriceps, a 15% muscle loss reaches a critical threshold point, leading to persistent fibrotic and inflammatory responses, as well as incomplete innervation and partial myofiber regeneration [195]. Additionally, the anatomical location of the muscle, such as the abdominal wall, latissimus dorsi (LD), TA, and quadriceps muscles, can further impact the potential for VML treatment, given the differing mechanical loading experienced by each [149]. To address these challenges, future efforts should prioritize the development of standardized protocols for creating VML injury models, encompassing factors such as anatomical location, muscle loading conditions, defect size and thickness, bridging versus non-bridging lesions, and the proportion of defect size relative to animal weight.

While various TE constructs for VML show promise, their success rates have varied due to distinct design factors. These factors include scaffold selection (hydrogels, 3D printed, aligned scaffolds, decellularized extracellular matrix), inclusion or exclusion of cells and their sources, use of growth factors, selection and processing of natural biomaterial sources, methods of decellularization and recellularization, application of electrical and mechanical stimulation, among others. Even within specific parameters, evaluating the success of TE constructs remains challenging due to procedural variations. For example, Passipieri et al. conducted a study where they harvested MDPCs from male Lewis rats and implanted them into female Lewis rats [185]. While this strategy aimed to minimize donor animals and simplify functional recovery evaluation, studies have shown sex-related variations in skeletal muscle fiber composition, gene expression, contractile function, and regenerative ability [196]. Moreover, there have been observations of improved regeneration efficiency of muscle stem cells derived from females in dystrophic mice [197]. This is consistent with documented sex differences in kidney transplants in mice, emphasizing the role of both implanted cell genotype and the host environment in gene expression during cross-sex implantation [198].

Lastly, the diversity of measures and criteria for evaluating tissue regeneration and functional recovery must be acknowledged. While commonly used histological staining provides semi-quantitative assessment of VML regeneration, including myotube and myofiber alignment, vascularization, and innervation. Quantitative measures such as muscle mass and isometric torque serve as standard indicators of functional recovery. However, it is recognized that force transmission, muscle recovery, muscle mass, and force generation do not exhibit perfect correlation. Normalizing these measures by tissue volume enhances their effectiveness as predictors of clinical relevance. Specifically, isometric torque density has emerged as a more effective clinical indicator than muscle mass [199]. Innovative approaches, such as volumetric MRI for evaluating reinnervation, are also being explored to identify methods with high clinical predictive values [200]. Notably, several clinical trials have recently evaluated muscle regeneration therapies [201], currently XENMATRIX AB is the sole treatment currently in clinical trials for VML [202]. The porcine collagen-based scaffold used in this trial had prior approval for applications in hernia repair and abdominal plastic and reconstructive surgery.

## Conclusion

TE skeletal muscle constructs have displayed remarkable potential in emulating the architecture and hierarchy of native tissue, leading to tissue regeneration and enhanced functional recovery. Furthermore, scaffolds that integrate spatiotemporal patterning of both biophysical and biochemical cues hold the promise of more accurately reproducing native skeletal muscle tissue, thereby likely promoting effective tissue regeneration. Clinical investigations primarily involve dECM-based scaffolds due to their advantages, including cost-effectiveness, simplicity, off-the-shelf availability, and expedited FDA approval processes. Although cell-based therapies are intricate, they yield superior outcomes. Progress toward achieving fully functional regeneration has been incremental, hampered by variability among studies and the relatively nascent state of the field. Future endeavors for treating VML should emphasize enhancing cell survival and engraftment at the injury site, facilitating the formation and maturation of aligned myotubes, and restoring vasculature and innervation.

## References

[1] Grogan, B. F., J. R. Hsu, Skeletal Trauma Research Consortium. Volumetric muscle loss. *J. Am. Acad. Orthop. Surg.* 19(Suppl 1):S35–S37, 2011. 21304045 10.5435/00124635-201102001-00007

[2] Sorensen, J. R., J. McFaline-Figueroa, and J. A. Call. Pathophysiology of volumetric muscle loss and targets for regenerative rehabilitation. In: Regenerative Rehabilitation: From Basic Science to the Clinic. Springer, 2022, pp. 177–225.

[3] Bartolacci, J. Scaffolds for skeletal muscle tissue engineering. In: Handbook of Tissue Engineering Scaffolds. 2019.

[4] Dolan, C. P., et al. The impact of bilateral injuries on the pathophysiology and functional outcomes of volumetric muscle loss. *npj Regen. Med.* 7(1):59, 2022. 36243737 10.1038/s41536-022-00255-2 PMC9569363

[5] Corona, B. T., et al. Volumetric muscle loss leads to permanent disability following extremity trauma. *J. Rehabil. Res. Dev.* 52(7):785–792, 2015. 26745661 10.1682/JRRD.2014.07.0165

[6] Corona, B. T., J. C. Wenke, and C. L. Ward. Pathophysiology of volumetric muscle loss injury. *Cells Tissues Organs*. 202(3–4):180–188, 2016. 27825160 10.1159/000443925

[7] Garg, K., et al. Volumetric muscle loss: persistent functional deficits beyond frank loss of tissue. *J. Orthop. Res.* 33(1):40–46, 2015. 25231205 10.1002/jor.22730

[8] Greising, S. M., C. L. Dearth, and B. T. Corona. Regenerative and rehabilitative medicine: a necessary synergy for functional recovery from volumetric muscle loss injury. *Cells Tissues Organs*. 202(3–4):237–249, 2016. 27825146 10.1159/000444673 PMC5553044

[9] Merrick, M. A. Secondary injury after musculoskeletal trauma: a review and update. *J. Athl. Train.* 37(2):209–217, 2002. 16558673 PMC164347

[10] Patel, K. H., et al. Aligned nanofibers of decellularized muscle extracellular matrix for volumetric muscle loss. *J. Biomed. Mater. Res. B*. 108(6):2528–2537, 2020. 10.1002/jbm.b.34584 32052931

[11] Roberts, K., et al. Transcriptome profiling of a synergistic volumetric muscle loss repair strategy. *BMC Musculoskelet. Disord.* 24(1):321, 2023. 37095469 10.1186/s12891-023-06401-1 PMC10124022

[12] Kiran, S., et al. Immunomodulation and biomaterials: key players to repair volumetric muscle loss. *Cells*. 10(8):2016, 2021. 34440785 10.3390/cells10082016 PMC8394423

[13] Hurtgen, B. J., et al. Severe muscle trauma triggers heightened and prolonged local musculoskeletal inflammation and impairs adjacent tibia fracture healing. *J. Musculoskelet. Neuronal Interact.* 16(2):122–134, 2016. 27282456 PMC5114355

[14] Hyldahl, R. D., et al. Extracellular matrix remodeling and its contribution to protective adaptation following lengthening contractions in human muscle. *FASEB J.* 29(7):2894–2904, 2015. 25808538 10.1096/fj.14-266668

[15] Sarrafian, T. L., et al. Extracellular matrix scaffolds for treatment of large volume muscle injuries: a review. *Vet. Surg.* 47(4):524–535, 2018. 29603757 10.1111/vsu.12787

[16] Sorensen, J. R., et al. Secondary denervation is a chronic pathophysiologic sequela of volumetric muscle loss. *J. Appl. Physiol.* 130(5):1614–1625, 2021. 33830817 10.1152/japplphysiol.00049.2021 PMC8354822

[17] Downing, K., et al. Old and new biomarkers for volumetric muscle loss. *Curr. Opin. Pharmacol.* 59:61–69, 2021. 34146835 10.1016/j.coph.2021.05.001 PMC8906174

[18] Doi, K., et al. Basic science behind functioning free muscle transplantation. *Clin. Plast. Surg.* 29(4):483–495, v–vi, 2002. 12484600 10.1016/S0094-1298(02)00020-2

[19] Kobayashi, S., et al. Functioning free muscle transplantation to the lower leg. *J. Reconstr. Microsurg.* 11(5):319–325, 1995. 8568737 10.1055/s-2007-1006546

[20] Lin, C. H., et al. Free functioning muscle transfer for lower extremity posttraumatic composite structure and functional defect. *Plast. Reconstr. Surg.* 119(7):2118–2126, 2007. 17519710 10.1097/01.prs.0000260595.85557.41

[21] Testa, S., et al. The war after war: volumetric muscle loss incidence, implication, current therapies and emerging reconstructive strategies, a comprehensive review. *Biomedicines*. 9(5):564, 2021. 34069964 10.3390/biomedicines9050564 PMC8157822

[22] Owens, J. G., et al. Return to running and sports participation after limb salvage. *J. Trauma*. 71(1 Suppl):S120–S124, 2011. 21795870 10.1097/TA.0b013e3182219225

[23] Aurora, A., et al. Physical rehabilitation improves muscle function following volumetric muscle loss injury. *BMC Sports Sci. Med. Rehabil.* 6(1):41, 2014. 25598983 10.1186/2052-1847-6-41 PMC4297368

[24] Washington, T. A., et al. The effect of autologous repair and voluntary wheel running on force recovery in a rat model of volumetric muscle loss. *Exp. Physiol.* 106(4):994–1004, 2021. 33600045 10.1113/EP089207 PMC8628541

[25] Nuge, T., et al. Recent advances in scaffolding from natural-based polymers for volumetric muscle injury. *Molecules*. 26(3):699, 2021. 33572728 10.3390/molecules26030699 PMC7865392

[26] Rodriguez, B. L., et al. A comparison of ovine facial and limb muscle as a primary cell source for engineered skeletal muscle. *Tissue Eng. A*. 26(3–4):167–177, 2020. 10.1089/ten.tea.2019.0087 PMC7044784 31469044

[27] Carnes, M. E., and G. D. Pins. Skeletal muscle tissue engineering: biomaterials-based strategies for the treatment of volumetric muscle loss. *Bioengineering (Basel)*. 7(3):85, 2020. 32751847 10.3390/bioengineering7030085 PMC7552659

[28] Langridge, B., M. Griffin, and P. E. Butler. Regenerative medicine for skeletal muscle loss: a review of current tissue engineering approaches. *J. Mater. Sci. Mater. Med.* 32(1):15, 2021. 33475855 10.1007/s10856-020-06476-5 PMC7819922

[29] Engler, A. J., et al. Myotubes differentiate optimally on substrates with tissue-like stiffness: pathological implications for soft or stiff microenvironments. *J. Cell Biol.* 166(6):877–887, 2004. 15364962 10.1083/jcb.200405004 PMC2172122

[30] Eugenis, I., D. Wu, and T. A. Rando. Cells, scaffolds, and bioactive factors: engineering strategies for improving regeneration following volumetric muscle loss. *Biomaterials*.278:121173, 2021. 34619561 10.1016/j.biomaterials.2021.121173 PMC8556323

[31] Boontheekul, T., et al. Regulating myoblast phenotype through controlled gel stiffness and degradation. *Tissue Eng.* 13(7):1431–1442, 2007. 17561804 10.1089/ten.2006.0356

[32] Cezar, C. A., and D. J. Mooney. Biomaterial-based delivery for skeletal muscle repair. *Adv. Drug Deliv. Rev.* 84:188–197, 2015. 25271446 10.1016/j.addr.2014.09.008 PMC4377112

[33] Causa, F., P. A. Netti, and L. Ambrosio. A multi-functional scaffold for tissue regeneration: the need to engineer a tissue analogue. *Biomaterials*. 28(34):5093–5099, 2007. 17675151 10.1016/j.biomaterials.2007.07.030

[34] Hench, L. L., and J. M. Polak. Third-generation biomedical materials. *Science*. 295(5557):1014–1017, 2002. 11834817 10.1126/science.1067404

[35] Dong, R., P. X. Ma, and B. Guo. Conductive biomaterials for muscle tissue engineering. *Biomaterials*.229:119584, 2020. 31704468 10.1016/j.biomaterials.2019.119584

[36] Pantelic, M. N., and L. M. Larkin. Stem cells for skeletal muscle tissue engineering. *Tissue Eng. B*. 24(5):373–391, 2018. 10.1089/ten.teb.2017.0451 29652595

[37] Sicari, B. M., C. L. Dearth, and S. F. Badylak. Tissue engineering and regenerative medicine approaches to enhance the functional response to skeletal muscle injury. *Anat. Rec. (Hoboken)*. 297(1):51–64, 2014. 24293290 10.1002/ar.22794

[38] Grasman, J. M., et al. Biomimetic scaffolds for regeneration of volumetric muscle loss in skeletal muscle injuries. *Acta Biomater.* 25:2–15, 2015. 26219862 10.1016/j.actbio.2015.07.038 PMC4562809

[39] Rouger, K., et al. Muscle satellite cell heterogeneity: in vitro and in vivo evidences for populations that fuse differently. *Cell Tissue Res.* 317(3):319–326, 2004. 15322909 10.1007/s00441-004-0911-9

[40] Montarras, D., et al. Direct isolation of satellite cells for skeletal muscle regeneration. *Science*. 309(5743):2064–2067, 2005. 16141372 10.1126/science.1114758

[41] Nakayama, K. H., et al. Treatment of volumetric muscle loss in mice using nanofibrillar scaffolds enhances vascular organization and integration. *Commun. Biol.* 2:170, 2019. 31098403 10.1038/s42003-019-0416-4 PMC6505043

[42] Machingal, M. A., et al. A tissue-engineered muscle repair construct for functional restoration of an irrecoverable muscle injury in a murine model. *Tissue Eng. A*. 17(17–18):2291–2303, 2011. 10.1089/ten.tea.2010.0682 PMC3161107 21548710

[43] Woodbury, D., et al. Adult rat and human bone marrow stromal cells differentiate into neurons. *J. Neurosci. Res.* 61(4):364–370, 2000. 10931522 10.1002/1097-4547(20000815)61:4<364::AID-JNR2>3.0.CO;2-C

[44] Pittenger, M. F., et al. Multilineage potential of adult human mesenchymal stem cells. *Science*. 284(5411):143–147, 1999. 10102814 10.1126/science.284.5411.143

[45] Strem, B. M., et al. Multipotential differentiation of adipose tissue-derived stem cells. *Keio J. Med.* 54(3):132–141, 2005. 16237275 10.2302/kjm.54.132

[46] Di Rocco, G., et al. Myogenic potential of adipose-tissue-derived cells. *J. Cell Sci.* 119(Pt 14):2945–2952, 2006. 16825428 10.1242/jcs.03029

[47] Matthias, N., et al. Volumetric muscle loss injury repair using in situ fibrin gel cast seeded with muscle-derived stem cells (MDSCs). *Stem Cell Res.* 27:65–73, 2018. 29331939 10.1016/j.scr.2018.01.008 PMC5851454

[48] Mizuno, Y., et al. Generation of skeletal muscle stem/progenitor cells from murine induced pluripotent stem cells. *FASEB J.* 24(7):2245–2253, 2010. 20181939 10.1096/fj.09-137174

[49] van der Wal, E., et al. Large-scale expansion of human iPSC-derived skeletal muscle cells for disease modeling and cell-based therapeutic strategies. *Stem Cell Rep.* 10(6):1975–1990, 2018. 10.1016/j.stemcr.2018.04.002 PMC5993675 29731431

[50] Iberite, F., E. Gruppioni, and L. Ricotti. Skeletal muscle differentiation of human iPSCs meets bioengineering strategies: perspectives and challenges. *npj Regen. Med.* 7(1):23, 2022. 35393412 10.1038/s41536-022-00216-9 PMC8991236

[51] Smoak, M. M., and A. G. Mikos. Advances in biomaterials for skeletal muscle engineering and obstacles still to overcome. *Mater. Today Bio*.7:100069, 2020. 32695987 10.1016/j.mtbio.2020.100069 PMC7363708

[52] Sicari, B. M., et al. An acellular biologic scaffold promotes skeletal muscle formation in mice and humans with volumetric muscle loss. *Sci. Transl. Med.* 6(234):234ra58, 2014. 24786326 10.1126/scitranslmed.3008085 PMC5942588

[53] Corona, B. T., and S. M. Greising. Challenges to acellular biological scaffold mediated skeletal muscle tissue regeneration. *Biomaterials*. 104:238–246, 2016. 27472161 10.1016/j.biomaterials.2016.07.020

[54] Qazi, T. H., et al. Biomaterials based strategies for skeletal muscle tissue engineering: existing technologies and future trends. *Biomaterials*. 53:502–521, 2015. 25890747 10.1016/j.biomaterials.2015.02.110

[55] Alarcin, E., et al. Current strategies for the regeneration of skeletal muscle tissue. *Int. J. Mol. Sci.* 22(11):5929, 2021. 34072959 10.3390/ijms22115929 PMC8198586

[56] Sicari, B. M., R. Londono, and S. F. Badylak. Strategies for skeletal muscle tissue engineering: seed vs. soil. *J. Mater. Chem. B*. 3(40):7881–7895, 2015. 32262901 10.1039/C5TB01714A

[57] Gilbert-Honick, J., and W. Grayson. Vascularized and innervated skeletal muscle tissue engineering. *Adv. Healthc. Mater.*9(1):e1900626, 2020. 31622051 10.1002/adhm.201900626 PMC6986325

[58] Das, S., et al. Pre-innervated tissue-engineered muscle promotes a pro-regenerative microenvironment following volumetric muscle loss. *Commun. Biol.* 3(1):330, 2020. 32587337 10.1038/s42003-020-1056-4 PMC7316777

[59] Badylak, S. F., D. O. Freytes, and T. W. Gilbert. Extracellular matrix as a biological scaffold material: structure and function. *Acta Biomater.* 5(1):1–13, 2009. 18938117 10.1016/j.actbio.2008.09.013

[60] Wolf, M. T., et al. Naturally derived and synthetic scaffolds for skeletal muscle reconstruction. *Adv. Drug Deliv. Rev.* 84:208–221, 2015. 25174309 10.1016/j.addr.2014.08.011 PMC5942587

[61] Reing, J. E., et al. Degradation products of extracellular matrix affect cell migration and proliferation. *Tissue Eng. A*. 15(3):605–614, 2009. 10.1089/ten.tea.2007.0425 18652541

[62] Sicari, B. M., et al. The promotion of a constructive macrophage phenotype by solubilized extracellular matrix. *Biomaterials*. 35(30):8605–8612, 2014. 25043569 10.1016/j.biomaterials.2014.06.060

[63] Choi, Y. J., et al. A 3D cell printed muscle construct with tissue-derived bioink for the treatment of volumetric muscle loss. *Biomaterials*. 206:160–169, 2019. 30939408 10.1016/j.biomaterials.2019.03.036

[64] Sicari, B. M., et al. A murine model of volumetric muscle loss and a regenerative medicine approach for tissue replacement. *Tissue Eng. A*. 18(19–20):1941–1948, 2012. 10.1089/ten.tea.2012.0475 PMC3463275 22906411

[65] Turner, N. J., et al. Xenogeneic extracellular matrix as an inductive scaffold for regeneration of a functioning musculotendinous junction. *Tissue Eng. A*. 16(11):3309–3317, 2010. 10.1089/ten.tea.2010.0169 20528669

[66] Valentin, J. E., et al. Functional skeletal muscle formation with a biologic scaffold. *Biomaterials*. 31(29):7475–7484, 2010. 20638716 10.1016/j.biomaterials.2010.06.039 PMC2922042

[67] Corona, B. T., et al. The promotion of a functional fibrosis in skeletal muscle with volumetric muscle loss injury following the transplantation of muscle-ECM. *Biomaterials*. 34(13):3324–3335, 2013. 23384793 10.1016/j.biomaterials.2013.01.061

[68] Garg, K., et al. Transplantation of devitalized muscle scaffolds is insufficient for appreciable de novo muscle fiber regeneration after volumetric muscle loss injury. *Cell Tissue Res.* 358(3):857–873, 2014. 25300647 10.1007/s00441-014-2006-6

[69] Aurora, A., et al. An acellular biologic scaffold does not regenerate appreciable de novo muscle tissue in rat models of volumetric muscle loss injury. *Biomaterials*. 67:393–407, 2015. 26256250 10.1016/j.biomaterials.2015.07.040

[70] Greising, S. M., et al. Unwavering pathobiology of volumetric muscle loss injury. *Sci. Rep.* 7(1):13179, 2017. 29030619 10.1038/s41598-017-13306-2 PMC5640632

[71] Mase, V. J., Jr., et al. Clinical application of an acellular biologic scaffold for surgical repair of a large, traumatic quadriceps femoris muscle defect. *Orthopedics*. 33(7):511, 2010. 20608620 10.3928/01477447-20100526-24

[72] Dziki, J., et al. An acellular biologic scaffold treatment for volumetric muscle loss: results of a 13-patient cohort study. *npj Regen. Med.* 1:16008, 2016. 29302336 10.1038/npjregenmed.2016.8 PMC5744714

[73] Conconi, M. T., et al. Homologous muscle acellular matrix seeded with autologous myoblasts as a tissue-engineering approach to abdominal wall-defect repair. *Biomaterials*. 26(15):2567–2574, 2005. 15585259 10.1016/j.biomaterials.2004.07.035

[74] Shin, T. H., et al. Human umbilical cord blood-stem cells direct macrophage polarization and block inflammasome activation to alleviate rheumatoid arthritis. *Cell Death Dis.*7(12):e2524, 2016. 28005072 10.1038/cddis.2016.442 PMC5260999

[75] Matziolis, G., et al. Autologous bone marrow-derived cells enhance muscle strength following skeletal muscle crush injury in rats. *Tissue Eng.* 12(2):361–367, 2006. 16548694 10.1089/ten.2006.12.361

[76] Qiu, X., et al. Mesenchymal stem cells and extracellular matrix scaffold promote muscle regeneration by synergistically regulating macrophage polarization toward the M2 phenotype. *Stem Cell Res. Ther.* 9(1):88, 2018. 29615126 10.1186/s13287-018-0821-5 PMC5883419

[77] Merritt, E. K., et al. Repair of traumatic skeletal muscle injury with bone-marrow-derived mesenchymal stem cells seeded on extracellular matrix. *Tissue Eng. A*. 16(9):2871–2881, 2010. 10.1089/ten.tea.2009.0826 20412030

[78] Winkler, T., et al. Dose-response relationship of mesenchymal stem cell transplantation and functional regeneration after severe skeletal muscle injury in rats. *Tissue Eng. A*. 15(3):487–492, 2009. 10.1089/ten.tea.2007.0426 18673090

[79] Carlson, B. M., and E. Gutmann. Development of contractile properties of minced muscle regenerates in the rat. *Exp. Neurol.* 36(2):239–249, 1972. 5053353 10.1016/0014-4886(72)90020-9

[80] Kasukonis, B., et al. Codelivery of infusion decellularized skeletal muscle with minced muscle autografts improved recovery from volumetric muscle loss injury in a rat model. *Tissue Eng. A*. 22(19–20):1151–1163, 2016. 10.1089/ten.tea.2016.0134 PMC5073241 27570911

[81] Goldman, S. M., and B. T. Corona. Co-delivery of micronized urinary bladder matrix damps regenerative capacity of minced muscle grafts in the treatment of volumetric muscle loss injuries. *PLoS ONE*.12(10):e0186593, 2017. 29040321 10.1371/journal.pone.0186593 PMC5645132

[82] Crapo, P. M., T. W. Gilbert, and S. F. Badylak. An overview of tissue and whole organ decellularization processes. *Biomaterials*. 32(12):3233–3243, 2011. 21296410 10.1016/j.biomaterials.2011.01.057 PMC3084613

[83] du Moon, G., et al. Cyclic mechanical preconditioning improves engineered muscle contraction. *Tissue Eng. A*. 14(4):473–482, 2008. 10.1089/tea.2007.0104 18399787

[84] Cheng, Y. W., et al. Engineering aligned skeletal muscle tissue using decellularized plant-derived scaffolds. *ACS Biomater. Sci. Eng.* 6(5):3046–3054, 2020. 33463300 10.1021/acsbiomaterials.0c00058 PMC8628848

[85] Xiao, S., et al. Gelatin methacrylate (GelMA)-based hydrogels for cell transplantation: an effective strategy for tissue engineering. *Stem Cell Rev. Rep.* 15(5):664–679, 2019. 31154619 10.1007/s12015-019-09893-4

[86] Duan, K., et al. Human iPSC-derived vascular smooth muscle cells in a fibronectin functionalized collagen hydrogel augment endothelial cell morphogenesis. *Bioengineering (Basel)*. 8(12):223, 2021. 34940376 10.3390/bioengineering8120223 PMC8698933

[87] Basurto, I. M., et al. Photoreactive hydrogel stiffness influences volumetric muscle loss repair. *Tissue Eng. A*. 28(7–8):312–329, 2022. 10.1089/ten.tea.2021.0137 PMC9057873 34409861

[88] DeQuach, J. A., et al. Injectable skeletal muscle matrix hydrogel promotes neovascularization and muscle cell infiltration in a hindlimb ischemia model. *Eur. Cell Mater.* 23:400–412, 2012. (**discussion 412**) 22665162 10.22203/eCM.v023a31 PMC3524267

[89] Marcinczyk, M., et al. The effect of laminin-111 hydrogels on muscle regeneration in a murine model of injury. *Tissue Eng. A*. 25(13–14):1001–1012, 2019. 10.1089/ten.tea.2018.0200 PMC9839345 30426851

[90] van Wachem, P. B., L. A. Brouwer, and M. J. van Luyn. Absence of muscle regeneration after implantation of a collagen matrix seeded with myoblasts. *Biomaterials*. 20(5):419–426, 1999. 10204984 10.1016/S0142-9612(98)00185-9

[91] Ward, C. L., L. Ji, and B. T. Corona. An autologous muscle tissue expansion approach for the treatment of volumetric muscle loss. *BioRes. Open Access*. 4(1):198–208, 2015. 26309796 10.1089/biores.2015.0009 PMC4497650

[92] Goldman, S. M., et al. Co-delivery of a laminin-111 supplemented hyaluronic acid based hydrogel with minced muscle graft in the treatment of volumetric muscle loss injury. *PLoS ONE*.13(1):e0191245, 2018. 29329332 10.1371/journal.pone.0191245 PMC5766229

[93] Pereira, T., et al. Effects of human mesenchymal stem cells isolated from Wharton’s jelly of the umbilical cord and conditioned media on skeletal muscle regeneration using a myectomy model. *Stem Cells Int.*2014:376918, 2014. 25379040 10.1155/2014/376918 PMC4212633

[94] Huang, H., et al. Preferred M2 polarization by ASC-based hydrogel accelerated angiogenesis and myogenesis in volumetric muscle loss rats. *Stem Cells Int.* 2017:2896874, 2017. 28694827 10.1155/2017/2896874 PMC5488492

[95] Beier, J. P., et al. Tissue engineering of injectable muscle: three-dimensional myoblast-fibrin injection in the syngeneic rat animal model. *Plast. Reconstr. Surg.* 118(5):1113–1121, 2006. 17016175 10.1097/01.prs.0000221007.97115.1d

[96] Rossi, C. A., et al. In vivo tissue engineering of functional skeletal muscle by freshly isolated satellite cells embedded in a photopolymerizable hydrogel. *FASEB J.* 25(7):2296–2304, 2011. 21450908 10.1096/fj.10-174755

[97] Wang, L., et al. Injectable remote magnetic nanofiber/hydrogel multiscale scaffold for functional anisotropic skeletal muscle regeneration. *Biomaterials*.285:121537, 2022. 35500394 10.1016/j.biomaterials.2022.121537

[98] Lee, W., et al. Thermosensitive hydrogel harboring CD146/IGF-1 nanoparticles for skeletal-muscle regeneration. *ACS Appl. Bio Mater.* 4(9):7070–7080, 2021. 35006939 10.1021/acsabm.1c00688

[99] Chen, C. S. Mechanotransduction—a field pulling together? *J. Cell Sci.* 121(Pt 20):3285–3292, 2008. 18843115 10.1242/jcs.023507

[100] Wells, R. G. The role of matrix stiffness in regulating cell behavior. *Hepatology*. 47(4):1394–1400, 2008. 18307210 10.1002/hep.22193

[101] Engler, A. J., et al. Matrix elasticity directs stem cell lineage specification. *Cell*. 126(4):677–689, 2006. 16923388 10.1016/j.cell.2006.06.044

[102] Ansari, S., et al. Muscle tissue engineering using gingival mesenchymal stem cells encapsulated in alginate hydrogels containing multiple growth factors. *Ann. Biomed. Eng.* 44(6):1908–1920, 2016. 27009085 10.1007/s10439-016-1594-6 PMC4880526

[103] Han, W. M., et al. Co-delivery of Wnt7a and muscle stem cells using synthetic bioadhesive hydrogel enhances murine muscle regeneration and cell migration during engraftment. *Acta Biomater.* 94:243–252, 2019. 31228633 10.1016/j.actbio.2019.06.025 PMC6642840

[104] Salimath, A. S., and A. J. Garcia. Biofunctional hydrogels for skeletal muscle constructs. *J. Tissue Eng. Regen. Med.* 10(11):967–976, 2016. 24616405 10.1002/term.1881

[105] Carleton, M. M., M. Locke, and M. V. Sefton. Methacrylic acid-based hydrogels enhance skeletal muscle regeneration after volumetric muscle loss in mice. *Biomaterials*.275:120909, 2021. 34087582 10.1016/j.biomaterials.2021.120909

[106] Ibanez-Fonseca, A., et al. Elastin-like recombinamer hydrogels for improved skeletal muscle healing through modulation of macrophage polarization. *Front. Bioeng. Biotechnol.* 8:413, 2020. 32478048 10.3389/fbioe.2020.00413 PMC7240013

[107] Harris, L. D., B. S. Kim, and D. J. Mooney. Open pore biodegradable matrices formed with gas foaming. *J. Biomed. Mater. Res.* 42(3):396–402, 1998. 9788501 10.1002/(SICI)1097-4636(19981205)42:3<396::AID-JBM7>3.0.CO;2-E

[108] Kin, S., et al. Regeneration of skeletal muscle using in situ tissue engineering on an acellular collagen sponge scaffold in a rabbit model. *ASAIO J.* 53(4):506–513, 2007. 17667240 10.1097/MAT.0b013e3180d09d81

[109] Haas, G. J., et al. Biomimetic sponges for regeneration of skeletal muscle following trauma. *J. Biomed. Mater. Res. A*. 107(1):92–103, 2019. 30394640 10.1002/jbm.a.36535

[110] Saxena, A. K., et al. Skeletal muscle tissue engineering using isolated myoblasts on synthetic biodegradable polymers: preliminary studies. *Tissue Eng.* 5(6):525–532, 1999. 10611544 10.1089/ten.1999.5.525

[111] Saxena, A. K., G. H. Willital, and J. P. Vacanti. Vascularized three-dimensional skeletal muscle tissue-engineering. *Biomed. Mater. Eng.* 11(4):275–281, 2001. 11790859

[112] Kamelger, F. S., et al. A comparative study of three different biomaterials in the engineering of skeletal muscle using a rat animal model. *Biomaterials*. 25(9):1649–1655, 2004. 14697866 10.1016/S0142-9612(03)00520-9

[113] Gillies, A. R., and R. L. Lieber. Structure and function of the skeletal muscle extracellular matrix. *Muscle Nerve*. 44(3):318–331, 2011. 21949456 10.1002/mus.22094 PMC3177172

[114] Beier, J. P., et al. Collagen matrices from sponge to nano: new perspectives for tissue engineering of skeletal muscle. *BMC Biotechnol.* 9:34, 2009. 19368709 10.1186/1472-6750-9-34 PMC2674407

[115] Huang, N. F., R. J. Lee, and S. Li. Engineering of aligned skeletal muscle by micropatterning. *Am. J. Transl. Res.* 2(1):43–55, 2010. 20182581 PMC2826821

[116] Lam, M. T., et al. Microfeature guided skeletal muscle tissue engineering for highly organized 3-dimensional free-standing constructs. *Biomaterials*. 30(6):1150–1155, 2009. 19064284 10.1016/j.biomaterials.2008.11.014

[117] Charest, J. L., A. J. Garcia, and W. P. King. Myoblast alignment and differentiation on cell culture substrates with microscale topography and model chemistries. *Biomaterials*. 28(13):2202–2210, 2007. 17267031 10.1016/j.biomaterials.2007.01.020

[118] Wang, P. Y., H. T. Yu, and W. B. Tsai. Modulation of alignment and differentiation of skeletal myoblasts by submicron ridges/grooves surface structure. *Biotechnol. Bioeng.* 106(2):285–294, 2010. 20148416 10.1002/bit.22697

[119] Huang, N. F., et al. Myotube assembly on nanofibrous and micropatterned polymers. *Nano Lett.* 6(3):537–542, 2006. 16522058 10.1021/nl060060o

[120] Metavarayuth, K., et al. Influence of surface topographical cues on the differentiation of mesenchymal stem cells in vitro. *ACS Biomater. Sci. Eng.* 2(2):142–151, 2016. 33418629 10.1021/acsbiomaterials.5b00377

[121] Sensini, A., and L. Cristofolini. Biofabrication of electrospun scaffolds for the regeneration of tendons and ligaments. *Materials (Basel)*. 11(10):1963, 2018. 30322082 10.3390/ma11101963 PMC6213815

[122] Wang, S.-X., et al. Electrospinning: a facile technique for fabricating functional nanofibers for environmental applications. *Nanotechnol. Rev.* 2016. 10.1515/ntrev-2015-0065. 10.1515/ntrev-2015-0065

[123] Tebyetekerwa, M., and S. Ramakrishna. What is next for electrospinning? *Matter*. 2(2):279–283, 2020. 10.1016/j.matt.2020.01.004

[124] Nakayama, K. H., et al. Rehabilitative exercise and spatially patterned nanofibrillar scaffolds enhance vascularization and innervation following volumetric muscle loss. *npj Regen. Med.* 3:16, 2018. 30245849 10.1038/s41536-018-0054-3 PMC6141593

[125] Choi, J. S., et al. The influence of electrospun aligned poly(epsilon-caprolactone)/collagen nanofiber meshes on the formation of self-aligned skeletal muscle myotubes. *Biomaterials*. 29(19):2899–2906, 2008. 18400295 10.1016/j.biomaterials.2008.03.031

[126] Beldjilali-Labro, M., et al. Multiscale-engineered muscle constructs: PEG hydrogel micro-patterning on an electrospun PCL Mat functionalized with gold nanoparticles. *Int. J. Mol. Sci.* 23(1):260, 2021. 35008686 10.3390/ijms23010260 PMC8745500

[127] Guo, Y., et al. Modified cell-electrospinning for 3D myogenesis of C2C12s in aligned fibrin microfiber bundles. *Biochem. Biophys. Res. Commun.* 516(2):558–564, 2019. 31235253 10.1016/j.bbrc.2019.06.082

[128] Gilbert-Honick, J., et al. Adipose-derived stem/stromal cells on electrospun fibrin microfiber bundles enable moderate muscle reconstruction in a volumetric muscle loss model. *Cell Transplant.* 27(11):1644–1656, 2018. 30298751 10.1177/0963689718805370 PMC6299198

[129] Gilbert-Honick, J., et al. Engineering functional and histological regeneration of vascularized skeletal muscle. *Biomaterials*. 164:70–79, 2018. 29499437 10.1016/j.biomaterials.2018.02.006

[130] Smoak, M. M., et al. Fabrication and characterization of electrospun decellularized muscle-derived scaffolds. *Tissue Eng. C*. 25(5):276–287, 2019. 10.1089/ten.tec.2018.0339 PMC6535957 30909819

[131] Patel, K. H., et al. Aligned nanofibers of decellularized muscle ECM support myogenic activity in primary satellite cells in vitro. *Biomed. Mater.*14(3):035010, 2019. 30812025 10.1088/1748-605X/ab0b06

[132] Smoak, M. M., et al. Bioinspired electrospun dECM scaffolds guide cell growth and control the formation of myotubes. *Sci. Adv.* 2021. 10.1126/sciadv.abg4123. 33990336 10.1126/sciadv.abg4123 PMC8121426

[133] Grasman, J. M., et al. Crosslinking strategies facilitate tunable structural properties of fibrin microthreads. *Acta Biomater.* 8(11):4020–4030, 2012. 22824528 10.1016/j.actbio.2012.07.018

[134] O’Brien, M. P., et al. Designing biopolymer microthreads for tissue engineering and regenerative medicine. *Curr. Stem Cell Rep.* 2(2):147–157, 2016. 27642550 10.1007/s40778-016-0041-9 PMC5019572

[135] Cornwell, K. G., and G. D. Pins. Discrete crosslinked fibrin microthread scaffolds for tissue regeneration. *J. Biomed. Mater. Res. A*. 82(1):104–112, 2007. 17269139 10.1002/jbm.a.31057

[136] Page, R. L., et al. Restoration of skeletal muscle defects with adult human cells delivered on fibrin microthreads. *Tissue Eng. A*. 17(21–22):2629–2640, 2011. 10.1089/ten.tea.2011.0024 21699414

[137] Kroehne, V., et al. Use of a novel collagen matrix with oriented pore structure for muscle cell differentiation in cell culture and in grafts. *J. Cell. Mol. Med.* 12(5A):1640–1648, 2008. 18194451 10.1111/j.1582-4934.2008.00238.x PMC2680279

[138] Jana, S., A. Cooper, and M. Zhang. Chitosan scaffolds with unidirectional microtubular pores for large skeletal myotube generation. *Adv. Healthc. Mater.* 2(4):557–561, 2013. 23184507 10.1002/adhm.201200177

[139] Rangarajan, S., L. Madden, and N. Bursac. Use of flow, electrical, and mechanical stimulation to promote engineering of striated muscles. *Ann. Biomed. Eng.* 42(7):1391–1405, 2014. 24366526 10.1007/s10439-013-0966-4 PMC4069203

[140] Herbison, G. J., M. M. Jaweed, and J. F. Ditunno. Muscle atrophy in rats following denervation, casting, inflammation, and tenotomy. *Arch. Phys. Med. Rehabil.* 60(9):401–404, 1979. 496606

[141] Kim, H., M. C. Kim, and H. H. Asada. Extracellular matrix remodelling induced by alternating electrical and mechanical stimulations increases the contraction of engineered skeletal muscle tissues. *Sci. Rep.* 9(1):2732, 2019. 30804393 10.1038/s41598-019-39522-6 PMC6389954

[142] Serena, E., et al. Electrophysiologic stimulation improves myogenic potential of muscle precursor cells grown in a 3D collagen scaffold. *Neurol. Res.* 30(2):207–214, 2008. 18397614 10.1179/174313208X281109

[143] Aparicio-Collado, J. L., et al. Pro-myogenic environment promoted by the synergistic effect of conductive polymer nanocomposites combined with extracellular zinc ions. *Biology (Basel)*. 11(12):1706, 2022. 36552216 10.3390/biology11121706 PMC9774464

[144] Basturkmen, B., et al. Silver nanowire loaded poly(epsilon-caprolactone) nanocomposite fibers as electroactive scaffolds for skeletal muscle regeneration. *Biomater. Adv.*134:112567, 2022. 35527139 10.1016/j.msec.2021.112567

[145] Jeong, S. I., et al. Development of electroactive and elastic nanofibers that contain polyaniline and poly(l-lactide-co-epsilon-caprolactone) for the control of cell adhesion. *Macromol. Biosci.* 8(7):627–637, 2008. 18401867 10.1002/mabi.200800005

[146] Chen, M. C., Y. C. Sun, and Y. H. Chen. Electrically conductive nanofibers with highly oriented structures and their potential application in skeletal muscle tissue engineering. *Acta Biomater.* 9(3):5562–5572, 2013. 23099301 10.1016/j.actbio.2012.10.024

[147] Hosseinzadeh, S., et al. The nanofibrous PAN-PANi scaffold as an efficient substrate for skeletal muscle differentiation using satellite cells. *Bioprocess Biosyst. Eng.* 39(7):1163–1172, 2016. 27086138 10.1007/s00449-016-1592-y

[148] Velasco-Mallorqui, F., et al. New volumetric CNT-doped gelatin-cellulose scaffolds for skeletal muscle tissue engineering. *Nanoscale Adv.* 2(7):2885–2896, 2020. 36132391 10.1039/D0NA00268B PMC9418820

[149] Sasaki, M., et al. Highly conductive stretchable and biocompatible electrode-hydrogel hybrids for advanced tissue engineering. *Adv. Healthc. Mater.* 3(11):1919–1927, 2014. 24912988 10.1002/adhm.201400209

[150] Ahadian, S., et al. Hydrogels containing metallic glass sub-micron wires for regulating skeletal muscle cell behaviour. *Biomater. Sci.* 3(11):1449–1458, 2015. 26343776 10.1039/C5BM00215J

[151] Grossi, A., K. Yadav, and M. A. Lawson. Mechanical stimulation increases proliferation, differentiation and protein expression in culture: stimulation effects are substrate dependent. *J. Biomech.* 40(15):3354–3362, 2007. 17582421 10.1016/j.jbiomech.2007.05.007

[152] Passipieri, J. A., et al. In silico and in vivo studies detect functional repair mechanisms in a volumetric muscle loss injury. *Tissue Eng. A*. 25(17–18):1272–1288, 2019. 10.1089/ten.tea.2018.0280 PMC6760186 30882277

[153] Corona, B. T., et al. Further development of a tissue engineered muscle repair construct in vitro for enhanced functional recovery following implantation in vivo in a murine model of volumetric muscle loss injury. *Tissue Eng. A*. 18(11–12):1213–1228, 2012. 10.1089/ten.tea.2011.0614 PMC3360508 22439962

[154] Corona, B. T., et al. Implantation of in vitro tissue engineered muscle repair constructs and bladder acellular matrices partially restore in vivo skeletal muscle function in a rat model of volumetric muscle loss injury. *Tissue Eng. A*. 20(3–4):705–715, 2014. 10.1089/ten.tea.2012.0761 PMC4518882 24066899

[155] Mintz, E. L., et al. Long-term evaluation of functional outcomes following rat volumetric muscle loss injury and repair. *Tissue Eng. A*. 26(3–4):140–156, 2020. 10.1089/ten.tea.2019.0126 PMC7047124 31578935

[156] Gholobova, D., et al. Endothelial network formation within human tissue-engineered skeletal muscle. *Tissue Eng. A*. 21(19–20):2548–2558, 2015. 10.1089/ten.tea.2015.0093 PMC4605445 26177063

[157] Juhas, M., et al. Biomimetic engineered muscle with capacity for vascular integration and functional maturation in vivo. *Proc. Natl. Acad. Sci. U. S. A.* 111(15):5508–5513, 2014. 24706792 10.1073/pnas.1402723111 PMC3992675

[158] Liao, I. C., et al. Effect of electromechanical stimulation on the maturation of myotubes on aligned electrospun fibers. *Cell. Mol. Bioeng.* 1(2–3):133–145, 2008. 19774099 10.1007/s12195-008-0021-y PMC2747747

[159] Husmann, I., et al. Growth factors in skeletal muscle regeneration. *Cytokine Growth Factor Rev.* 7(3):249–258, 1996. 8971480 10.1016/S1359-6101(96)00029-9

[160] Tatsumi, R., et al. HGF/SF is present in normal adult skeletal muscle and is capable of activating satellite cells. *Dev. Biol.* 194(1):114–128, 1998. 9473336 10.1006/dbio.1997.8803

[161] Xin, X., et al. Hepatocyte growth factor enhances vascular endothelial growth factor-induced angiogenesis in vitro and in vivo. *Am. J. Pathol.* 158(3):1111–1120, 2001. 11238059 10.1016/S0002-9440(10)64058-8 PMC1850376

[162] Yablonka-Reuveni, Z., R. Seger, and A. J. Rivera. Fibroblast growth factor promotes recruitment of skeletal muscle satellite cells in young and old rats. *J. Histochem. Cytochem.* 47(1):23–42, 1999. 9857210 10.1177/002215549904700104

[163] Kang, W. F., and J. M. Hébert. FGF signaling is necessary for neurogenesis in young mice and sufficient to reverse its decline in old mice. *J. Neurosci.* 35(28):10217–10223, 2015. 26180198 10.1523/JNEUROSCI.1469-15.2015 PMC4502262

[164] Oladipupo, S. S., et al. Endothelial cell FGF signaling is required for injury response but not for vascular homeostasis. *Proc. Natl. Acad. Sci. U. S. A.* 111(37):13379–13384, 2014. 25139991 10.1073/pnas.1324235111 PMC4169958

[165] Chakravarthy, M. V., et al. Insulin-like growth factor-I extends replicative life span of skeletal muscle satellite cells by enhancing G/S cell cycle progression via the activation of phosphatidylinositol 3′-kinase/Akt signaling pathway. *J. Biol. Chem.* 275(46):35942–35952, 2000. 10962000 10.1074/jbc.M005832200

[166] Chargé, S. B. P., and M. A. Rudnicki. Cellular and molecular regulation of muscle regeneration. *Physiol. Rev.* 84(1):209–238, 2004. 14715915 10.1152/physrev.00019.2003

[167] Gardner, S., et al. Separating myoblast differentiation from muscle cell fusion using IGF-I and the p38 MAP kinase inhibitor SB202190. *Am. J. Physiol. Cell Physiol.* 309(7):C491-500, 2015. 26246429 10.1152/ajpcell.00184.2015 PMC4593770

[168] Alcazar, C. A., et al. Transplantation of insulin-like growth factor-1 laden scaffolds combined with exercise promotes neuroregeneration and angiogenesis in a preclinical muscle injury model. *Biomater. Sci.* 8(19):5376–5389, 2020. 32996916 10.1039/D0BM00990C PMC7531607

[169] Pelosi, L., et al. Local expression of IGF-1 accelerates muscle regeneration by rapidly modulating inflammatory cytokines and chemokines. *FASEB J.* 21(7):1393–1402, 2007. 17264161 10.1096/fj.06-7690com

[170] Borselli, C., et al. The role of multifunctional delivery scaffold in the ability of cultured myoblasts to promote muscle regeneration. *Biomaterials*. 32(34):8905–8914, 2011. 21911253 10.1016/j.biomaterials.2011.08.019 PMC3210474

[171] Arsic, N., et al. Vascular endothelial growth factor stimulates skeletal muscle regeneration. *Mol. Ther.* 10(5):844–854, 2004. 15509502 10.1016/j.ymthe.2004.08.007

[172] Ferrara, N. Role of vascular endothelial growth factor in physiologic and pathologic angiogenesis: therapeutic implications. *Semin. Oncol.* 29(6):10–14, 2002. 12516033 10.1016/S0093-7754(02)70064-X

[173] Allen, R. E., and L. K. Boxhorn. Regulation of skeletal muscle satellite cell proliferation and differentiation by transforming growth factor-beta, insulin-like growth factor I, and fibroblast growth factor. *J. Cell. Physiol.* 138(2):311–315, 1989. 2918032 10.1002/jcp.1041380213

[174] Weist, M. R., et al. TGF-beta1 enhances contractility in engineered skeletal muscle. *J. Tissue Eng. Regen. Med.* 7(7):562–571, 2013. 22371337 10.1002/term.551 PMC4035810

[175] Rathbone, C. R., et al. Effects of transforming growth factor-beta (TGF-β1) on satellite cell activation and survival during oxidative stress. *J. Muscle Res. Cell Motil.* 32(2):99–109, 2011. 21823037 10.1007/s10974-011-9255-8

[176] Hamaguchi, H., et al. PDGF-B secreted from skeletal muscle enhances myoblast proliferation and myotube maturation via activation of the PDGFR signaling cascade. *Biochem. Biophys. Res. Commun.* 639:169–175, 2023. 36521377 10.1016/j.bbrc.2022.11.085

[177] Hagiwara, K., et al. Promotion of muscle regeneration by myoblast transplantation combined with the controlled and sustained release of bFGFcpr. *J. Tissue Eng. Regen. Med.* 10(4):325–333, 2016. 23554408 10.1002/term.1732

[178] Hu, C., et al. Comparative effects of basic fibroblast growth factor delivery or voluntary exercise on muscle regeneration after volumetric muscle loss. *Bioengineering (Basel)*. 9(1):37, 2022. 35049746 10.3390/bioengineering9010037 PMC8773127

[179] Grasman, J. M., et al. Rapid release of growth factors regenerates force output in volumetric muscle loss injuries. *Biomaterials*. 72:49–60, 2015. 26344363 10.1016/j.biomaterials.2015.08.047 PMC4591244

[180] Lee, J., et al. Active blood vessel formation in the ischemic hindlimb mouse model using a microsphere/hydrogel combination system. *Pharm. Res.* 27(5):767–774, 2010. 20221675 10.1007/s11095-010-0067-0

[181] Shvartsman, D., et al. Sustained delivery of VEGF maintains innervation and promotes reperfusion in ischemic skeletal muscles via NGF/GDNF signaling. *Mol. Ther.* 22(7):1243–1253, 2014. 24769910 10.1038/mt.2014.76 PMC4089004

[182] Carmeliet, P., and M. Tessier-Lavigne. Common mechanisms of nerve and blood vessel wiring. *Nature*. 436(7048):193–200, 2005. 16015319 10.1038/nature03875

[183] Wang, L., et al. Minimally invasive approach to the repair of injured skeletal muscle with a shape-memory scaffold. *Mol. Ther.* 22(8):1441–1449, 2014. 24769909 10.1038/mt.2014.78 PMC4435589

[184] Pumberger, M., et al. Synthetic niche to modulate regenerative potential of MSCs and enhance skeletal muscle regeneration. *Biomaterials*. 99:95–108, 2016. 27235995 10.1016/j.biomaterials.2016.05.009

[185] Passipieri, J. A., et al. Keratin hydrogel enhances in vivo skeletal muscle function in a rat model of volumetric muscle loss. *Tissue Eng. A*. 23(11–12):556–571, 2017. 10.1089/ten.tea.2016.0458 PMC6916122 28169594

[186] Baker, H. B., et al. Cell and growth factor-loaded keratin hydrogels for treatment of volumetric muscle loss in a mouse model. *Tissue Eng. A*. 23(11–12):572–584, 2017. 10.1089/ten.tea.2016.0457 PMC6916118 28162053

[187] Hill, E., T. Boontheekul, and D. J. Mooney. Regulating activation of transplanted cells controls tissue regeneration. *Proc. Natl. Acad. Sci. U. S. A.* 103(8):2494–2499, 2006. 16477029 10.1073/pnas.0506004103 PMC1413770

[188] San Emeterio, C. L., et al. Nanofiber-based delivery of bioactive lipids promotes pro-regenerative inflammation and enhances muscle fiber growth after volumetric muscle loss. *Front. Bioeng. Biotechnol.*9:650289, 2021. 33816455 10.3389/fbioe.2021.650289 PMC8017294

[189] Pandolfi, L., et al. Composite microsphere-functionalized scaffold for the controlled release of small molecules in tissue engineering. *J. Tissue Eng.* 7:2041731415624668, 2016. 26977286 10.1177/2041731415624668 PMC4765809

[190] Blaho, V. A., and T. Hla. Regulation of mammalian physiology, development, and disease by the sphingosine 1-phosphate and lysophosphatidic acid receptors. *Chem. Rev.* 111(10):6299–6320, 2011. 21939239 10.1021/cr200273u PMC3216694

[191] Haas, G., et al. Biomimetic sponges improve muscle structure and function following volumetric muscle loss. *J. Biomed. Mater. Res. A*. 109(11):2280–2293, 2021. 33960118 10.1002/jbm.a.37212 PMC9838030

[192] Dumont, F. J. FK506, an immunosuppressant targeting calcineurin function. *Curr. Med. Chem.* 7(7):731–748, 2000. 10702636 10.2174/0929867003374723

[193] Larouche, J., et al. Robust inflammatory and fibrotic signaling following volumetric muscle loss: a barrier to muscle regeneration. *Cell Death Discov.* 9(3):409, 2018. 10.1038/s41419-018-0455-7 PMC5851980 29540673

[194] Aguilar, C. A., et al. Multiscale analysis of a regenerative therapy for treatment of volumetric muscle loss injury. *Cell Death Discov.* 4:33, 2018. 29531830 10.1038/s41420-018-0027-8 PMC5841404

[195] Anderson, S. E., et al. Determination of a critical size threshold for volumetric muscle loss in the mouse quadriceps. *Tissue Eng. C*. 25(2):59–70, 2019. 10.1089/ten.tec.2018.0324 PMC6389771 30648479

[196] Deasy, B. M., R. C. Schugar, and J. Huard. Sex differences in muscle-derived stem cells and skeletal muscle. *Crit. Rev. Eukaryot. Gene Expr.* 18(2):173–188, 2008. 18304031 10.1615/CritRevEukarGeneExpr.v18.i2.60

[197] Deasy, B. M., et al. A role for cell sex in stem cell-mediated skeletal muscle regeneration: female cells have higher muscle regeneration efficiency. *J. Cell Biol.* 177(1):73–86, 2007. 17420291 10.1083/jcb.200612094 PMC2064113

[198] Wang, L., et al. Cross-sex transplantation alters gene expression and enhances inflammatory response in the transplanted kidneys. *Am. J. Physiol. Ren. Physiol.* 313(2):F326–F338, 2017. 10.1152/ajprenal.00039.2017 PMC5582902 28515172

[199] Wang, L., et al. Muscle density, but not size, correlates well with muscle strength and physical performance. *J. Am. Med. Dir. Assoc.* 22(4):751-759 e2, 2021. 32768372 10.1016/j.jamda.2020.06.052

[200] Wilcox, M., et al. Volumetric MRI is a promising outcome measure of muscle reinnervation. *Sci. Rep.* 11(1):22433, 2021. 34789795 10.1038/s41598-021-01342-y PMC8599480

[201] Mulbauer, G. D., and H. W. T. Matthew. Biomimetic scaffolds in skeletal muscle regeneration. *Discoveries (Craiova)*.7(1):e90, 2019. 32309608 10.15190/d.2019.3 PMC7086065

[202] Enhanced Bioscaffold for Volumetric Muscle Loss. https://classic.clinicaltrials.gov/show/NCT04051242.

